# Remote Sensing of Depth-Induced Variations in Soil Organic Carbon Stocks Distribution Within Different Vegetated Landscapes

**DOI:** 10.1016/j.catena.2024.108216

**Published:** 2024-08

**Authors:** Omosalewa Odebiri, Onisimo Mutanga, John Odindi, Rob Slotow, Paramu Mafongoya, Romano Lottering, Rowan Naicker, Trylee Nyasha Matongera, Mthembeni Mngadi

**Affiliations:** 1 School of Agricultural, Earth and Environmental Sciences, Discipline of Geography, University of KwaZulu-Natal, Pietermaritzburg, South Africa; 2 Centre for Integrative Ecology, School of Life and Environmental Sciences, Deakin University, Melbourne, VIC 3125, Australia; 3 Centre for Transformative Agriculture and Food Systems, University of KwaZulu-Natal, Pietermaritzburg, South Africa; 4 Oppenheimer Fellow in Functional Biodiversity, Centre for Functional Biodiversity, School of Life Sciences, University of Kwazulu-Natal, Pietermaritzburg, South Africa; 5 Department of Genetics, Evolution and Environment, University College London, United Kingdom; 6 Agronomy and Rural Development, School of Agricultural, Earth and Environmental Sciences, University of KwaZulu-Natal, Scottsville, Pietermaritzburg, South Africa

**Keywords:** Soil organic carbon, Soil Depth, Land cover, Deep learning, Soil Management

## Abstract

The preservation and augmentation of soil organic carbon (SOC) stocks is critical to designing climate change mitigation strategies and alleviating global warming. However, due to the susceptibility of SOC stocks to environmental and topo-climatic variability and changes, it is essential to obtain a comprehensive understanding of the state of current SOC stocks both spatially and vertically. Consequently, to effectively assess SOC storage and sequestration capacity, precise evaluations at multiple soil depths are required. Hence, this study implemented an advanced Deep Neural Network (DNN) model incorporating Sentinel-1 Synthetic Aperture Radar (SAR) data, topo-climatic features, and soil physical properties to predict SOC stocks at multiple depths (0-30cm, 30-60cm, 60-100cm, and 100-200cm) across diverse land-use categories in the KwaZulu-Natal province, South Africa. There was a general decline in the accuracy of the DNN model’s prediction with increasing soil depth, with the root mean square error (RMSE) ranging from 8.34 t/h to 11.97 t/h for the four depths. These findings imply that the link between environmental covariates and SOC stocks weakens with soil depth. Additionally, distinct factors driving SOC stocks were discovered in both topsoil and deep-soil, with vegetation having the strongest effect in topsoil, and topo-climate factors and soil physical properties becoming more important as depth increases. This underscores the importance of incorporating depth-related soil properties in SOC modelling. Grasslands had the largest SOC stocks, while commercial forests have the highest SOC sequestration rates per unit area. This study offers valuable insights to policymakers and provides a basis for devising regional management strategies that can be used to effectively mitigate climate change.

## 1 Introduction

A recent report by the Intergovernmental Panel on Climate Change (IPCC) has sparked worldwide concern surrounding the detrimental impact of cumulative carbon emissions on global warming (Allen *et al*., 2019, Pörtner *et al*., 2022, Mariappan *et al*., 2023). To reach the 1.5 ºC target threshold advocated for by the IPCC, global climate interests have shifted towards a concurrent reduction in carbon emissions and prioritization of significant carbon storage reservoirs (Harper *et al*., 2018, Fuhrman *et al*., 2020, Wu *et al*., 2022). Amongst these efforts, soil organic carbon (SOC), which represents one of the largest terrestrial carbon pools, has emerged as an important mechanism for carbon storage and sequestration (Kenye *et al*., 2019, Sahoo *et al*., 2019, Li *et al*., 2021c). Besides climate change mitigation, SOC aids the retention of water and nutrients, thereby facilitating microbial activity and regulating overall soil fertility and functioning (Lee *et al*., 2019, Odebiri *et al*., 2023a). However, fluctuations in these stocks have the potential to disrupt the global carbon cycle and influence vegetation growth through variations in the soil biological and physicochemical properties (Xu *et al*., 2013, Sainepo *et al*., 2018, Lamichhane *et al*., 2022). Commonly, site-specific factors such as climate, topography, land-use, and soil type are known to influence SOC distribution and storage (Wiesmeier *et al*., 2019, Venter *et al*., 2021, Wang *et al*., 2021a). However, SOC is often not uniformly distributed amongst the soil profile, and the depth at which SOC is stored can impact soil health and productivity (Jandl *et al*., 2014).

Soil is typically categorized by a series of horizons, each with unique characteristics that affect its function and nutrient cycling (Eilers *et al*., 2012, Osman and Osman, 2013, Kalev and Toor, 2018). The subsurface soil horizon is divided into three distinct layers; the topsoil (0-10 cm), the subsoil (10-30 cm), and the substratum (>30 cm) (Eilers *et al*., 2012, Osman and Osman, 2013, Kalev and Toor, 2018). The topsoil, also known as the A horizon, is widely regarded as the most biologically active layer and often contains the highest concentration of organic matter, nutrients and microorganisms (Eilers *et al*., 2012), while the subsoil layer or B horizon, is typically considered less biologically active than the topsoil, but can still contain a significant amount of SOC (Osman and Osman, 2013). The substratum or C horizon is the deepest layer and is generally thought to contain the least amount of SOC (Avery, 1973, Eilers *et al*., 2012). Nevertheless, knowledge of SOC variability within the sub-soil and its dynamics remains relatively limited, as existing studies have mainly focused on the topsoil’s SOC accumulation (Hobley and Wilson, 2016). According to Rumpel *et al*. (2012) and Ngo *et al*. (2013), subsoil horizons may contribute to more than half of the total soil carbon stocks, hence should be integrated into global carbon estimates. In this regard, a comprehensive understanding of the vertical distribution of SOC within the soil profile, and its variation across different land-uses, is necessary for accurate prediction of SOC stocks, evaluation of soil fertility, and formulation of strategic management initiatives for climate change mitigation and enhanced soil health (Wang *et al*., 2016). Although literature (Don *et al*., 2011, Song *et al*., 2020, Li *et al*., 2023, Lin *et al*., 2023) has explored SOC at different depths, there is a general lack of consensus surrounding the dynamics of SOC storage at deeper depths (Lorenz and Lal, 2014, Scharlemann *et al*., 2014). Furthermore, little is known about the influence of environmental factors (such as climate, vegetation, soil type, mineral composition, soil texture, and topography) on regional SOC variability and storage at greater soil depths (Saiz *et al*., 2012, Zhuo *et al*., 2022). This understanding is further constrained by the variable nature of these factors, which can differ among sites and are often subject to continuous change in both space and time (Van Der Sande *et al*., 2023). Consequently, there is a large degree of uncertainty surrounding the relative significance and interactions of the different mechanisms that regulate SOC storage across diverse land-uses at various soil depths (Odebiri *et al*., 2020). Thus, given the evolving nature of present-day land-use change, it is imperative to regularly consider the effects of biotic and abiotic factors on SOC stocks at deeper soil depths across diverse land-uses (Zanella *et al*., 2018, Briassoulis, 2020), necessary for providing valuable insights into the factors governing the long-term storage and cycling of SOC within the soil profile.

Remote sensing technologies have become increasingly popular in recent years for estimating SOC stocks due to their non-destructive nature and large spatial coverage that enable efficient periodic SOC measurements (Wang *et al*., 2018, Nayak *et al*., 2019, Zhang *et al*., 2020, Odebiri *et al*., 2023a). However, different sensors and platforms have yielded varying degrees of success in SOC estimation across diverse environments (Odebiri *et al*., 2023b). For example, Ayala Izurieta *et al*. (2022) and Vaudour *et al*. (2019b) used Sentinel-2 imagery (400-2500nm) to estimate SOC at different soil depths, achieving R^2^ values that ranged from 0.58 to 0.86. Whereas multispectral sensors such as Landsat and Sentinel-2 are widely implemented for regional-scale SOC mapping, they have limited spectral resolution and can be susceptible to atmospheric interference leading to decreased accuracy (Taghizadeh-Mehrjardi *et al*., 2020). Alternatively, hyperspectral sensors such as AVIRIS, HyMap, and PRISMA have higher spectral resolutions and can capture reflectance in hundreds of narrow bands (Odebiri *et al*., 2021). For instance, Angelopoulou *et al*. (2023) utilized both Hypex and PRISMA hyperspectral data to estimate soil organic matter (SOM) in Northern Greece, obtaining R^2^ values of 0.79 and 0.76, respectively. However, hyperspectral sensors are often constrained by cost and a limited spatial coverage, rendering them impractical for large-scale applications (Yang *et al*., 2019).

Although optical remote sensing technology has been effective in estimating SOC, the emergence of platforms and sensors such as LiDAR and Synthetic Aperture Radar (e.g. Sentinel-1) has provided promising alternatives (Odebiri *et al*., 2021, Tripathi and Tiwari, 2022). These technologies offer distinct advantages, such as improved coverage and penetration through vegetation, as well as medium to high-resolution elevation data, making them a viable and attractive option for SOC estimation (Odebiri *et al*., 2021). These attributes are crucial for accurately estimating SOC, given that depth and volume are key factors within this process. Studies such as Shafizadeh-Moghadam *et al*. (2022) and Sothe *et al*. (2022) have demonstrated the potential of SAR data in estimating SOC across various depths. However, while remote sensing has been proven effective in predicting topsoil SOC, subsoil SOC prediction remains largely unexplored due to challenges in direct measurement and limited data for validation. Therefore, further research is needed to assess the effectiveness of these methods across different soil types and regions.

To address this research gap, this study sought to evaluate the effectiveness of different environmental variables and spectral indices in predicting subsoil SOC at various depths, whilst simultaneously identifying important factors that may influence this relationship. Through the incorporation of Sentinel-1 and SOC legacy data, this research sought to provide a comprehensive understanding of depth-induced variations in SOC stock distribution. Specifically, we sought to estimate SOC stock variability at four different depths (0-30 cm, 30-60 cm, 60-100 cm, and 100-200cm) across four different vegetated landscapes, namely natural forests, commercial forest plantations, croplands, and grasslands, using SAR imagery (Sentinel-1), and a deep learning approach.

## 2 Methodology

### 2.1 Study site description

**Figure 1:**
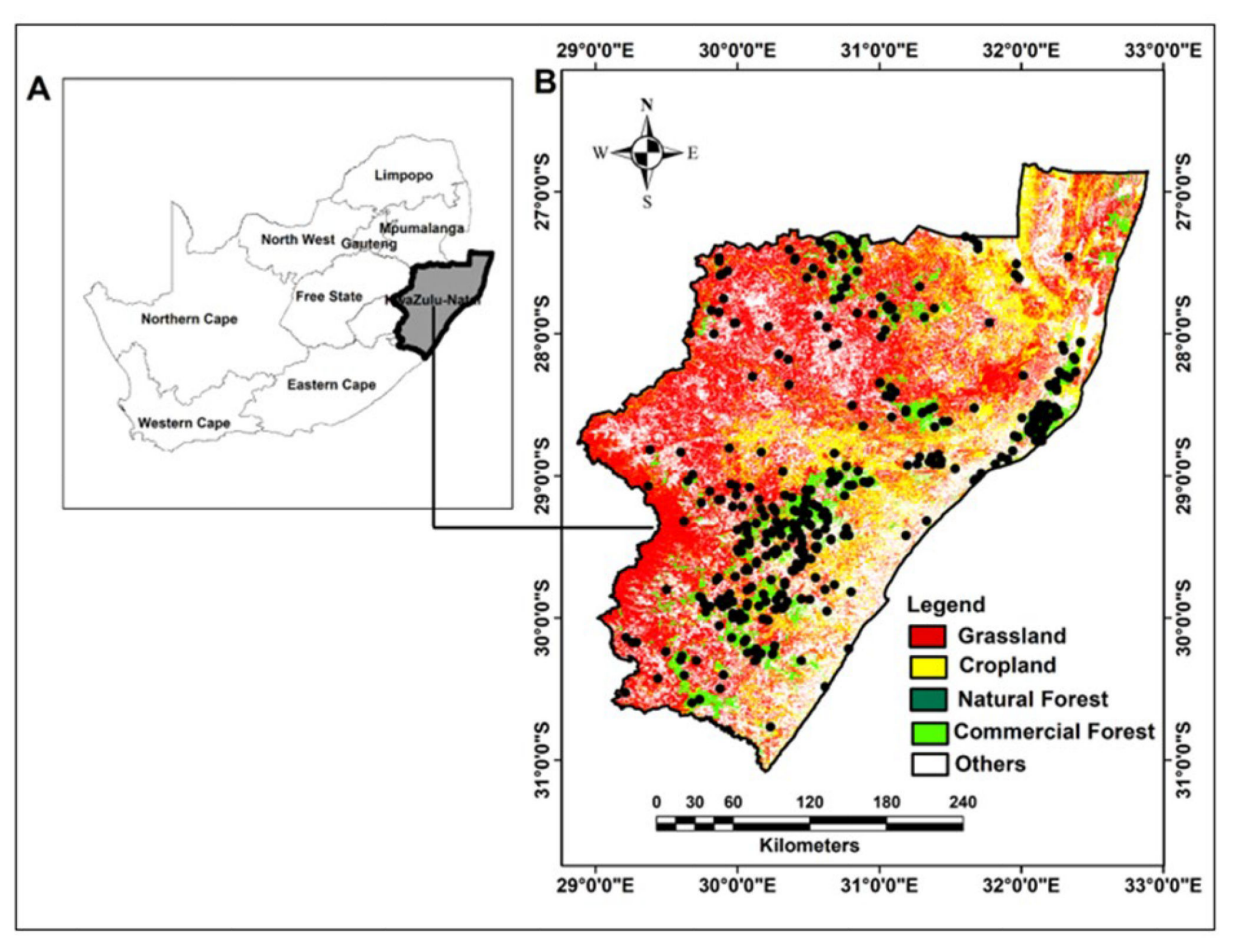
The location of KwaZulu-Natal province in South Africa (A) with (B) showing the spatial spread of soil samples (black dots) superimposed across the four vegetation types.

This study focused on the KwaZulu-Natal (KZN) province, located in the South Africa’s eastern seaboard (Figure 1) and covers approximately 94,000 km^2^ (Garnas *et al*., 2016). The province has a subtropical climate characterized by high levels of rainfall and warm temperatures, with mean annual temperature ranging from 15°C to 25°C and an average annual rainfall of approximately 1000 mm (Ndlovu *et al*., 2021, Mashao *et al*., 2023). The region has a rich diversity of vegetation types, including tropical and subtropical forests, grasslands, and savanna (Mucina and Rutherford, 2006). The province’s vegetation profile is dominated by a savanna with Acacia species, thicket, grasslands and forest ecosystems. The region also exhibits a diverse topography, with elevation ranging from sea level to approximately 3,482 meters above sea level (Carbutt, 2019). The province is underlain by different rock formations that include sandstone, shale, and limestone (Norman, 2013, Nell and Van Huyssteen, 2014). The sandstone formations are largely composed of quartz, feldspar, and lithic fragments, whereas the shale formations contain clay minerals and organic matter (Mucina and Rutherford, 2006). The limestone formations are mainly composed of calcium carbonate and exhibit karst topography. The region has a diverse range of soil types due to its varied topography, climate, and geology, with six dominant soil types found at different depths. These soil types include the coastal sandy soils, acidic soils, clay soils, sandy loam soils, rocky soils, and alluvial soils (Mucina and Rutherford, 2006, Fey, 2010). Coastal sandy soils have low organic carbon content due to high leaching and low nutrient retention, whereas acidic soils are characterized by high acidity and low soil organic carbon content (Mucina and Rutherford, 2006, Fey, 2010). Clay soils have high nutrient retention and high soil organic carbon content, while sandy loam soils have moderate nutrient retention and soil organic carbon content (Mucina and Rutherford, 2006, Fey, 2010). Rocky soils have been documented to have a low soil organic carbon content due to high erosion rates and low nutrient retention, while alluvial soils have both high nutrient and soil organic carbon content (Mucina and Rutherford, 2006, Fey, 2010). Overall, the biophysical characteristics of KZN, including its climate, soils, topography, and geology, play a crucial role in shaping the region’s diverse ecological systems and landscapes.

#### 2.1.2 Vegetative landscape description

This study investigates the SOC concentrations across four major vegetation types in KZN, covering over 84% of the province’s landscape: Natural Forests, Commercial Forests, Grasslands, and Croplands. Natural Forests, known for their rich biodiversity including species like Yellowwood (*Podocarpus spp*.) and Natal mahogany (*Trichilia emetica*), contribute to high SOC levels due to complex soil structures and species diversity (Gush et al., 2015; Fraser, 2012). Commercial Forests, dominated by exotic species like Eucalyptus for timber, show SOC fluctuations due to management practices (Dovey, 2014; Louw, 2016; Merino et al., 2004). Grasslands (both natural and managed), featuring a variety of forbs, grasses, and scattered trees, support carbon accumulation through extensive root systems and are vital for livestock grazing (Mucina and Rutherford, 2006; Blair et al., 2014; Matthews et al., 2001; Mbaabu et al., 2020; Ghosh and Mahanta, 2014). Croplands, mainly cultivated with crops like maize and sugarcane, exhibit SOC variations with intensive farming practices affecting subsoil SOC levels (Hitayezu et al., 2016; Vågen et al., 2005; Tiefenbacher et al., 2021). The unique diversity of ecosystems and specific climatic conditions (such as high levels of rainfall) found in the province can significantly influence SOC levels, highlighting the need to explore potential variations in SOC stocks at different depths across these regions.

### 2.2 Soil data

This investigation analysed 707 soil profiles, providing critical insights into the SOC content and bulk density at varying depths (0-30cm, 30-60cm, 60-100cm, 100-200cm) in KZN. Among these profiles, 407 were sourced from the International Soil Reference Information Centre (ISRIC), a non-profit organization that provide global, high-quality information on soil properties, including SOC. The remaining 300 points were obtained from previous soil investigations conducted by the Department of Agricultural Earth and Environmental Sciences (SAEES) at the University of KwaZulu-Natal. The ISRIC soil database, last updated in June 2022 (https://www.isric.org/), with over 200,000 sample points from 173 countries, incorporates different methods for determining SOC content and acquisition times and locations (Batjes *et al*., 2020). The ISRIC dataset has included corrections for stoniness within the calculations of bulk density (Batjes and van Oostrum, 2023, Grossman and Reinsch, 2002). To standardize the data, ISRIC developed harmonized procedures for uniform soil profile data input (Hengl *et al*., 2017, Venter *et al*., 2021), publicly available via their website (https://www.isric.org/explore/wosis/accessing-wosis-derived-datasets). In addition to the soil data from SAEES, the sample points covering the KZN Province, and their equivalent SOC content were obtained from the ISRIC database. Formula (1) below, devised by Pearson (2007), was employed to calculate the SOC stocks at various depths for each point.

(1) SOCstock=H×BD×OC×10

Where, *H* is thickness of horizon (cm); *BD*, bulk density (g cm^-2^); *OC*, soil organic carbon concentration in bulk soil (g kg^-1^)

### 2.3 Image data acquisition

#### 2.3.1 Sentinel-1 Data

The study utilized Sentinel-1 synthetic aperture radar (SAR) satellite data from the European Space Agency (ESA) under the European Union’s Copernicus Programme. Launched on April 3, 2014 (Peter *et al*., 2017), the Sentinel-1 is an active remote sensing system that transmits microwave pulses to the ground and measures the strength and phase of the returning signal, which enables it to penetrate through clouds and vegetation (Rodríguez-Veiga *et al*., 2017, Babaeian *et al*., 2019). This attribute, along with its ability to operate continuously day and night, makes it suitable for mapping different soil properties, including SOC and terrain features (Wang *et al*., 2021b). Sentinel-1 has three imaging modes; the interferometric wide swath (IW) mode, which has a spatial resolution of 5 meters and covers a swath width of 250 km, the strip map (SM) mode with a spatial resolution of 5 meters and a swath width of 80 km, and the extra-wide swath (EW) mode, with a spatial resolution of 20 meters and a swath width of 400 km (Nagler *et al*., 2015, Kim and Han, 2023). In this study, SAR images were adopted due to their superior capacity to map SOC stocks at greater depths. This is primarily attributed to their substantial penetrative power beyond the topsoil, which exceeds that of optical data. Notably, despite their potential, SAR images have seldom been adopted in digital soil mapping (Zhou *et al*., 2023).

The datasets were downloaded and pre-processed in the Google Earth Engine (GEE) platform to produce SAR images for both VV and VH polarizations (Table 1). To pre-process the Sentinel-1 data, the images were first imported into GEE and then filtered using the filter function to include only those with ascending orbits and VV and VH polarizations. Since the soil data used in this study is a legacy dataset that spans several years, the images were also filtered to include the median image of every image collection available from 2014 to the present. To remove the effects of topography, terrain correction was applied to the filtered Sentinel-1 data. Subsequently, smoothening was applied to the terrain-corrected images using the image-focal-median function with a window size of 5 pixels. To eliminate the noise prevalent in radar data, the smoothed images were subjected to speckle filtering. Specifically, the Refined Lee Speckle Filter, with a window size of 3 pixels, was applied separately for VV and VH polarizations (Qiu *et al*., 2004, Singh *et al*., 2021). A median reduction function was applied to the speckle-filtered images to create composite images for both VV and VH polarizations and stacked together into a single image for ease of analysis and visualization. Finally, a Sentinel-1 Radar Vegetation Index (RVI) was generated using the expression: 4*VH/(VV+VH), which represents an alternative to the Normalised Difference Vegetation Index (NDVI), with low vegetated or bare areas indicating low RVI while densely vegetated areas indicating higher RVI values (Mandal *et al*., 2020). The images were then exported at 20m resolution and used for further analysis within the Python environment.

#### 2.3.2 Topo-climate metrics and soil physical properties

In this study, we utilized eight influential terrain metrics (Table 1) previously identified in Odebiri *et al*. (2023a) and Odebiri *et al*. (2023b). These metrics comprise the Topographic Wetness Index (TWI), Direct Insolation, Slope, General Curvature, Catchment Area, Profile Curvature, Aspect, and Elevation (Table 1). These metrics were derived from a Shuttle Radar Topography Mission (SRTM) Digital Elevation Model (DEM) using SAGA GIS (2.3.2) and ArcGIS Pro 2.8 software. To improve the analysis, mean temperature and rainfall data for province were incorporated from the Worldclim dataset (http://www.worlclim.org/), which span over three decades of climate data. This information includes average annual temperature and rainfall, as well as the wettest, driest, coldest, and hottest quarters and months of the year. Both the DEM and Worldclim datasets were resampled to match the spatial resolution of the Sentinel 1 data (20m) using the raster resample function in ArcGIS Pro 2.8 (Abera *et al*., 2022, Price, 2023). Additionally, we incorporated other physical soil properties that have a significant influence on SOC stocks distribution (Hengl *et al*., 2017, Batjes *et al*., 2020). These included: coarse fragments, clay content, sand content, silt content, and soil type (Table 1). The soil type data was obtained from the Food and Agricultural Organization (FAO) soil portal (https://www.fao.org/soils-portal/data-hub/soil-maps-and-databases/faounesco-soil-map-of-the-world/en/), while other soil properties were obtained from ISRIC using the GEE platform at four different depths (0-30cm, 30-60cm, 60-100cm, 100-200cm) to match our soil data.

### 2.4 The SOC Model

In this study, we utilized a Deep Neural Network (DNN) architecture to simultaneously model SOC stocks at four different depths (0-30cm, 30-60cm, 60-100cm, and 100-200cm). DNNs are a type of artificial neural network that consists of multiple layers of interconnected nodes. Each node in a layer receives inputs from the previous layer, performs a computation, and passes the output to the next layer (Emadi *et al*., 2020). The final layer of the DNN model produces the prediction for SOC stock content.

**Figure 2:**
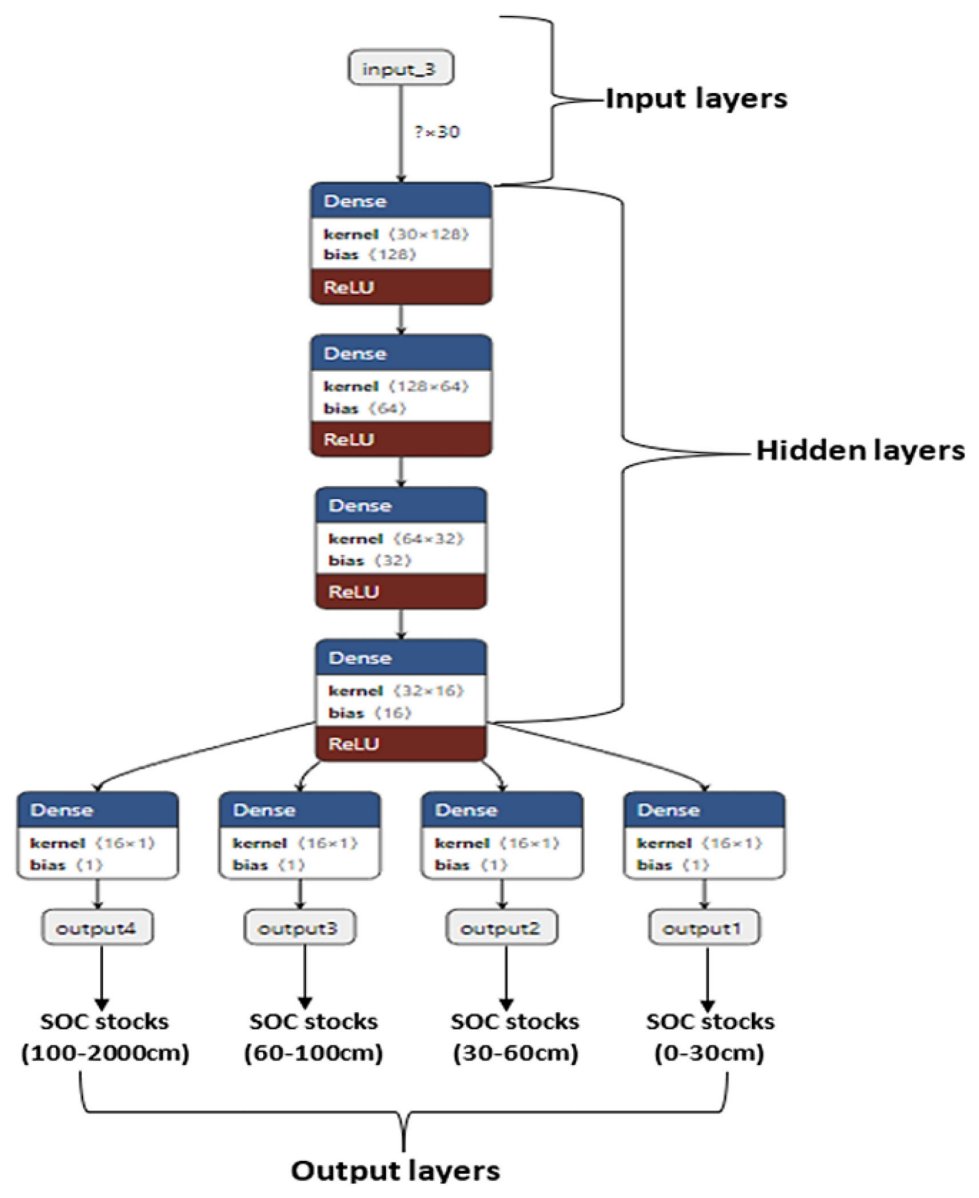
Deep neural network architecture with four output nodes for each soil depth. See text for details.

To achieve our objective, we used 18 input variables, which were standardized to have a mean of zero and a standard deviation of one, to ensure equal contributions to the model. We modified the architecture of the DNN model by adding four output nodes to the final layer, one for each depth, to produce four outputs simultaneously, that represent the predicted SOC stock for each depth. After performing hyper-parameter optimization using a train/validation/test split, and a 10-fold cross-validation (Odebiri *et al*., 2023b), the final model utilized four hidden layers and a rectified linear unit (ReLU) activation function (with 100 epochs, and a batch size of 32). Moreover, an Adam optimizer, with mean squared error as the loss function (which is standard for regression problems) was also implemented. To prevent overfitting, a dropout regularization was added after each hidden layer with a rate of 0.2. A linear activation function was used for the output layer, as predictions are continuous values. The model was trained with early stopping to prevent any additional overfitting. The analysis was performed using the Python programming language (version 3.8) within the Jupiter notebook environment. A mathematical representation of the model used in this study is provided in Figure 2 below, which shows the schematics of the model’s architecture. In summary, the DNN model employed in this study is a powerful analytical strategy for SOC mapping at different depths, as it can learn complex relationships between input variables and SOC stocks through a series of nonlinear transformations.

(2) y1,y2,y3,y4=f(W4f(W3f(W2f(W1x+b1)+b2)+b3)+b4).

where *x* is the input vector of covariates, *W1-W4* are the weight matrices of the four layers, *b1-b4* are the bias terms, and *f ()* is the activation function. The output variables *y1, y2, y3*, and *y4* represent the predicted SOC for the four different depths.

### 2.5 Model evaluation metrics

Three accuracy metrics, including the Root Mean Square Error (RMSE) and the Coefficient of Determination (R^2^) were used to evaluate the fitting and generalization of the models developed in this study. For the complete mathematical expressions of these metrics, see Odebiri *et al*. (2022b). Additionally, the usefulness of each covariate was evaluated to ascertain how much they contributed to SOC stocks variability at each depth. SHapely Additive exPlanations (SHAP) technique, a methodology for explaining the predictions of complex models, was adopted for this purpose. SHAP provides a unified approach to explainability that can be applied to a wide range of models and data types (Shapley, 1953). The key idea behind SHAP is to use Shapley values, a concept from cooperative game theory, to attribute the contribution of each feature to the prediction for a specific instance (Lundberg and Lee, 2017). SHAP can also be used to detect interactions and nonlinearity in the data, and to diagnose model failures and biases. For this study, a variant of the SHAP explainer (“DeepExplainer”) peculiar to deep learning models was implemented in “Jupiter notebooks” to generate importance rankings for the DNN model.

## 3 Results

### 3.1 Summary statistics of SOC data

Table 2 displays the descriptive statistics of the soil data (n=707) at each of the four distinct depths utilized in this study. Average SOC stocks in KZN ranged from 23.55t/h to 57.53 t/h. Interestingly, both average and maximum SOC stocks steadily decreased from a depth of 0-30 cm to 60-100 cm, however, once a depth greater than 1 metre was reached, SOC stocks increased considerably (Table 2). The topsoil data (0-30 cm) exhibited the lowest SOC stock variance (29%), while all other depths had a noticeably higher SOC variance (>40%). Furthermore, the SOC data displayed considerable skewness and kurtosis across all depths. A natural logarithm transformation (Log10) was subsequently implemented to generate new skewness and kurtosis values and provide a normal distribution of the data (Table 2). Thereafter, a predictive analysis was performed on the transformed SOC data, which was later transformed back to its original scale.

### 3.2 Model evaluation and performance at each soil depth

**Figure 3:**
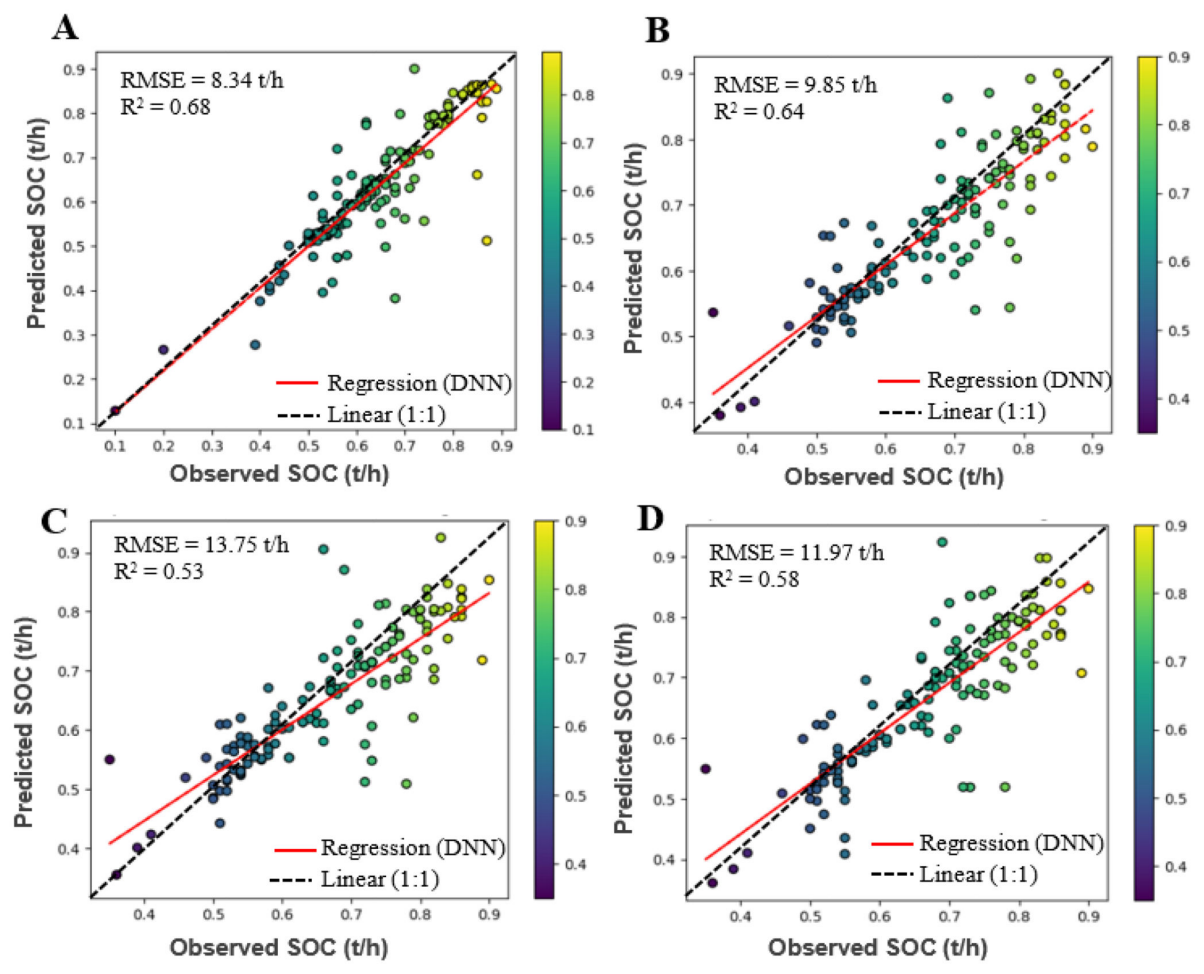
Scatter plots for test data with colour density representation of observed vs predicted SOC stocks, with A, B, C, and D denoting soil depths of 0-30 cm, 30-60 cm, 60-100 cm, and 100-200 cm, respectively.

Table 3 shows the performance of the DNN model across the four soil depths for SOC stocks predictions simultaneously for both the train and test data using 18 covariates that includes Sentinel 1 data, topo-climate and soil physical properties. For the 0-30cm depth (topsoil), the DNN model achieved an RMSE of 7.34t/h and an R^2^ of 0.74 for the train data, indicating that the model explained 74% of the variance in the data. For the test data, the RMSE increased to 8.34 t/h, but the R^2^ values remained high at 0.68. At the 30-60cm depth which signifies the start of the subsoil SOC stocks, the DNN model achieved an RMSE of 8.27 t/h and an R^2^ of 0.69 for the train data, indicating a moderate level of explanation of the data. For the test data, the RMSE increased to 9.85 t/h, and the R^2^ values decreased to 0.64. At the 60-100cm depth, the DNN model achieved an RMSE of 10.59 t/h and an R^2^ of 0.59 for the train data, indicating a lower level of explanation of the data compared to the first two depths. For the test data, the RMSE increased to 13.75 t/h, and the R^2^ values decreased to 0.53. At the 100-200cm depth, the DNN model slightly performed better than the third depth and achieved an RMSE of 10.05 t/h and an R^2^ of 0.61 for the train data, indicating a moderate level of explanation of the data. For the test data, the RMSE increased to 11.97 t/h, and the R^2^ values decreased to 0.58. The results (R^2^ and RMSE) depict a general reduction in evaluation and accuracy metrics as the soil depth increases, thus indicating that the interrelationships between the environmental covariates and SOC stocks decreases with increase in soil depths (Table 3). Figure 3 depicts the correlation between the observed and estimated SOC for the DNN model at each depth.

### 3.3 Assessment of variable importance at various soil depths

**Figure 4:**
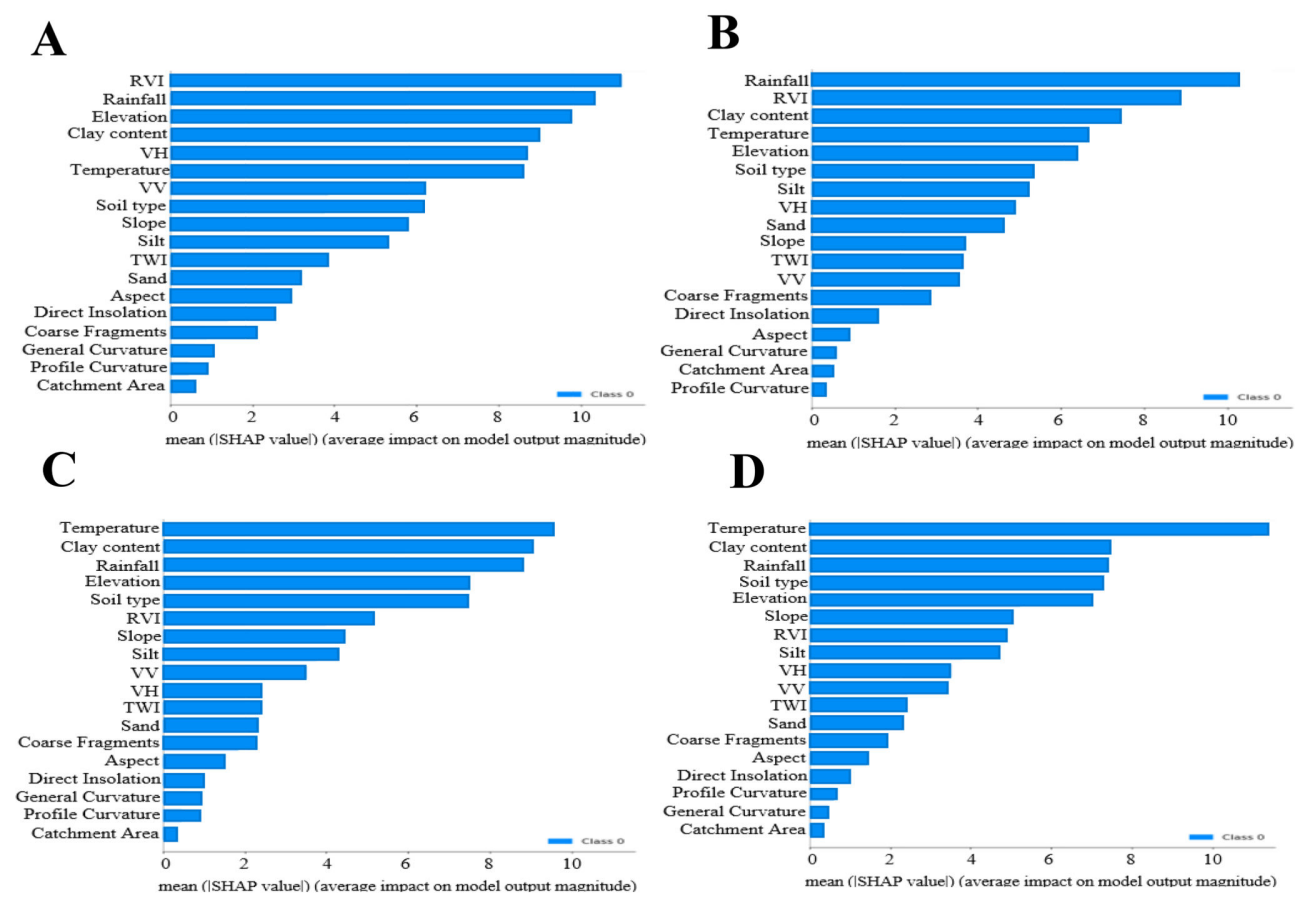
Variables importance ranking for SOC stocks distribution across four depths with A, B, C and D representing soil depths of 0-30 cm, 30-60 cm, 60-100 cm, and 100-200 cm, respectively.

The SHAP technique was used to rank the importance of the different covariates for predicting SOC stocks across the various soil depths (Figure 4). The analysis demonstrated a notable shift in the most important variables as the soil depth increased, indicating that the factors driving SOC stocks in the topsoil and deep soil are quite distinct. For the topsoil (0-30 cm), the five most important variables were RVI, rainfall, elevation, clay content, and VH. In contrast, for the second depth (30-60 cm), rainfall was the most important variable, followed by clay content, temperature, elevation, and RVI. For the third depth (60-100 cm), temperature, clay content, rainfall, elevation, and soil type were the most important. Finally, at the last depth (100-200 cm), temperature was again the most important variable, followed by clay content, rainfall, soil type, and elevation. Additionally, other variables such as silt, VV, TWI, sand, and slope also contributed significantly to the model across all depths, even though they were not ranked amongst the top five variables. Taken together, these findings emphasize the relative importance of different soil properties and environmental covariates on SOC stocks at different soil depths.

### 3.4 SOC storage potential across different soil depths and vegetation landscapes

The results showed that Grasslands ecosystem stored the largest amount of SOC across all depth ranges, representing 46.43% of the total SOC stocks, followed by Cropland, Natural Forest and Commercial Forest (Table 4). While Grasslands occupy the largest surface area, contributing significantly to the total SOC stocks, it’s important to note that Commercial Forest, despite covering a smaller area, accounts for a notable proportion of SOC stocks (10.47%). This suggests that Commercial Forest is particularly effective in SOC sequestration. The vertical distribution of SOC stocks also varied across land-uses, with Grasslands demonstrating the highest SOC stocks (16.29%) at a depth of 0-30 cm, which decreased considerably to 7.15% at a depth of 60-100 cm. Similarly, the 0-30 cm depth range showed the highest SOC stocks in Cropland, Natural Forest, and Commercial Forest. Meanwhile the 60-100 cm depth range had the lowest SOC stocks for each land-use type. Notably, once a depth of 1 metre was reached, SOC storage potential increased across each of the land-uses. In general, the results suggest that both land use and soil depth have a significant impact on Total SOC stocks.

Nevertheless, the study found that Grassland, Cropland, Natural Forest, and Commercial Forest all exhibited unique SOC storage potential at various soil depths. Specifically, at a depth of 0-30 cm, Commercial Forest and Natural Forest demonstrated the highest SOC storage potential (Table 4), followed by Grassland and Cropland. At 30-60 cm depth, Commercial Forest showed the highest SOC stocks, followed by Natural Forest, Grassland and Cropland. Similarly, at a depth of 60-100 cm, Commercial Forest showed the highest potential for SOC storage, followed by Natural Forest, Cropland and Grassland (Table 4). Finally, at a depth of 100-200 cm, Commercial Forest once again exhibited the highest SOC storage potential, followed by Grassland, Cropland and Natural Forest (Table 4). Overall, the results indicate that Commercial Forest had the highest mean SOC content across all depth intervals (Table 4). These findings underscore the significance of land-use type in determining SOC storage capacity and highlights the potential of strategic Commercial Forestry as an effective strategy for enhancing SOC stocks in KZN.

### 3.5 SOC geographical distribution across different soil depths and vegetation landscapes

**Figure 5:**
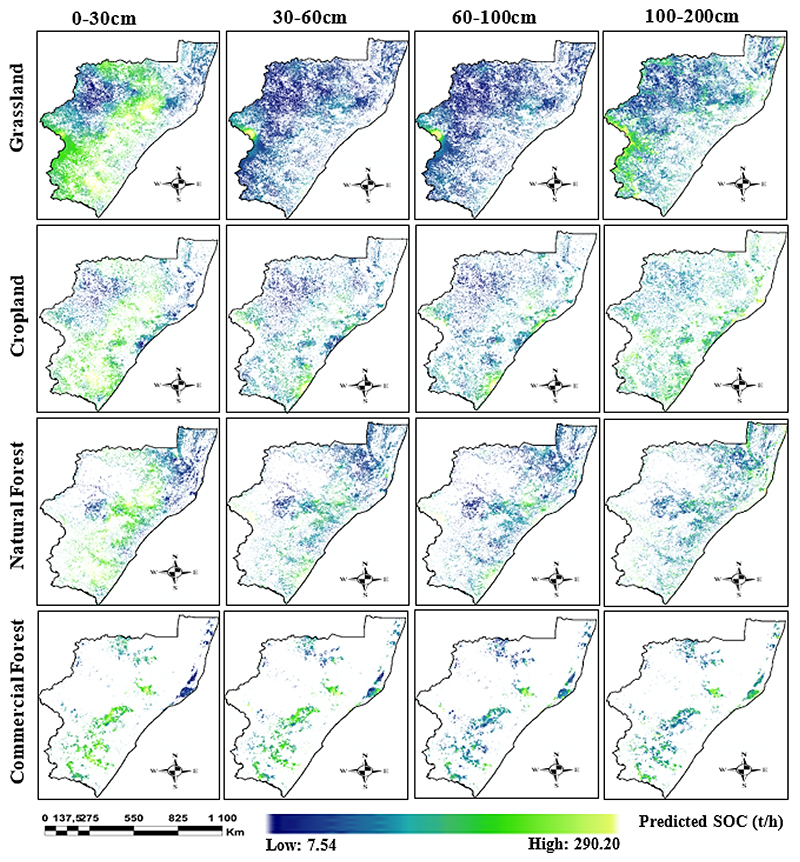
Distribution of Soil Organic Carbon (SOC) stocks across different soil depths and land uses in KwaZulu-Natal, South Africa. The SOC stocks are expressed in t/h for each land-use category (Grassland, Cropland, Natural Forest, and Commercial Forest) at for four different soil depth intervals (0-30cm, 30-60cm, 60-100cm, and 100-200cm).

The distribution of SOC stocks in the province exhibits a distinct pattern, with the majority of SOC stocks concentrated within the Southwestern edge of the province, extending along the central interior towards the Northen boundary (Figure 5). The SOC stocks for each land-use type are predominantly located at a soil depth of 0-30cm, with Grasslands having the highest amount of SOC, followed by Cropland, Natural Forest, and Commercial Forest (Table 4). In Grasslands, SOC stocks are concentrated along the Southwestern edge of KZN at the 0-30cm depth, but gradually decrease in concentration as the soil depth increases, shifting to the West of the province, close to the Drakensberg mountains. Similarly, Croplands have widely distributed SOC concentrations at the 0-30cm depth, but as the thickness of the soil horizon becomes deeper, SOC stocks become more abundant along the Southern coast, with isolated patches along the Northern coastal interior and Western regions (Figure 5). Natural Forests, however, display a strip-like distribution of SOC stocks along the coastal interior, with a cluster of SOC stocks located in the Central-Eastern part of the province. These stocks become more isolated towards the Central parts of the Eastern coastline as the soil depth increases. Finally, Commercial Forests have SOC stocks predominantly located in the Central and Southern parts of the province at the 0-30 cm depth, with isolated patches along the Northern regions (Figure 5). As the depth increases, the SOC stocks in Commercial Forests drastically diminish and become concentrated within isolated patches located along the Northern coastline, Southern regions, and the Central Midlands (Figure 5).

## 4 Discussion

### 4.1 Spatial and vertical distribution of SOC stocks across different vegetated landscapes within KwaZulu-Natal

The findings of this study indicate that substantial portions of KZN’s SOC are distributed across different soil depths, with approximately 35%, 19%, 15%, and 30% of SOC stored from 0–200 cm. Although the highest concentration of SOC was discovered in the topsoil, the deeper substratum layers (60-200cm) were found to store approximately 64% of the total SOC stocks. These results correspond with Batjes (2008) who reported a higher SOC stock value within Central African topsoil. This outcome is further supported by Chaopricha and Marín-Spiotta (2014) and Gross and Harrison (2019) but contradicts studies by Albaladejo *et al*. (2013) and Taghizadeh-Mehrjardi *et al*. (2016), who found that SOC was primarily located in the topsoil, with SOC concentrations decreasing with depth. According to literature, deep-rooted systems and bioturbation can contribute to the accumulation of SOC in subsoils (Hussain *et al*., 2021, Marín-Spiotta and Hobley, 2022). However, SOC composition can vary across different soil depths due to the erratic rates of plant biopolymer decomposition (Wang *et al*., 2021c). For instance, plant biopolymer substances, such as hemicellulose and pectin decompose faster than cellulose under aerobic conditions (López-Mondéjar et al., 2016, Hemati *et al*., 2022), resulting in variable rates of plant litter decomposition within the thickness of the soil horizon. Furthermore, the composition of subsoil carbon can be affected by the translocation of particulate and dissolved organic matter (Derenne and Largeau, 2001, Lorenz and Lal, 2005, Lorenz *et al*., 2007). Subsequently, the diversity and concentrations of available plant biopolymers and compounds, as well as their rates of decomposition, significantly influence SOC concentrations across diverse depths, which, in turn, can differ significantly among different land uses (Lorenz and Lal, 2005).

The findings show that Grasslands had the largest SOC stocks at all depths, accounting for approximately 46.43% of the total SOC stocks, with 35.11% stored within the topsoil. This is consistent with previous studies reporting larger SOC stocks in Grassland ecosystems (Lal, 2004, Kukal and Bawa, 2014, Chen *et al*., 2019). Grasslands, comprised of diverse grasses, graminoids, and forbs, are found along the Northern and Eastern interior of KZN (Smit *et al*., 1995, Palmer and Ainslie, 2005). Their large geographical extents mainly contribute SOC stocks in KZN (Fornara and Tilman, 2008). Additionally, diverse microbial communities in these ecosystems breaks down organic matter, releasing nutrients that benefit plant growth and stabilizing soil aggregates, improving soil structure and water-holding capacity at different depths (Farrell *et al*., 2020, Edwards and Arancon, 2022). Meanwhile, the region’s temperate and moist climate increases plant litter decomposition and improves SOC storage. Moreover, extensive root biomass structure enhances SOM, stabilizes soil, and promotes SOC within the subsoil structure (Bot and Benites, 2005, Mensah, 2015). This supports the study’s findings that grasslands store the majority of their SOC stocks (about 65%) below the topsoil (30-200cm), but contrasts a study by Lorenz and Lal (2005). Finally, the sandy composition of some soils in KZN (Dlamini *et al*., 2011) facilitates water and air movement, that promotes microbial activity, and facilitates SOC storage at different soil depths (Kalev and Toor, 2018).

Croplands in KZN, which occupy diverse soil types and terrain (Khumalo, 2016), accounted for 21.64% of the total SOC stocks, with the majority located in the topsoil. This corresponds to Assefa *et al*. (2017), Heikkinen *et al*. (2021), and Wiesmeier *et al*. (2014), who revealed higher carbon levels in the topsoil of agricultural landscapes. Although high temperatures and moisture levels in the region can reduce SOC storage by accelerating organic matter decomposition, the area’s ample rainfall and rich soils create favourable conditions for crop growth, leading to potential organic matter accumulation and SOC storage. While Croplands can enhance SOC storage through fertilizer application and soil cover (Follett, 2001), specific farming practices ultimately determine the level of SOC storage.

Forest’s landscapes, such as commercial and natural forests, showed smaller SOC stocks compared to grasslands and croplands due to their smaller geographical coverage, however, they store more SOC per unit area. In KZN, topsoil storage rates average 62.44 t/h and 57.27 t/h, respectively, consistent with previous studies highlighting forests’ significant storage accumulation potential (Paul *et al*., 2002, Jandl *et al*., 2007, Bárcena *et al*., 2014, Grüneberg *et al*., 2014). Commercial forests in KZN, particularly those with species like Eucalyptus and Pinus (Peerbhay *et al*., 2013), play a vital role in SOC storage (Lai, 2004). Mature Evergreen trees and Evergreen hardwoods contribute significantly to SOC accumulation, especially in deeper soils, aided by the deeper rooting zones (Marín-Spiotta and Sharma, 2013, Zhou *et al*., 2017). The deep rooting zone of Eucalyptus trees (Dell *et al*., 1983), also contributes to SOC storage capacities and corresponds to our findings within the deep soil. Sustainable management practices in commercial forests, such as erosion control and reduced tillage, can further enhance SOC storage across soil depths (Alemu, 2014, Lorenz and Lal, 2015). Natural forests in KZN, characterized by diverse evergreen trees, enhance SOC sequestration through litterfall and root turnover, leading to higher SOC levels (Lal, 2005, Odebiri *et al*., 2023a). High species diversity aids in nutrient cycling and carbon storage across soil depths, with different root systems affecting SOC distribution (Germon *et al*., 2020). Indigenous tree species, such as yellowwood (*Podocarpus spp*.) and Natal mahogany (*Trichilia emetica*) (Gush *et al*., 2015), support deeper SOC storage and soil quality through slow-decomposing litter and soil aggregation (Nair *et al*., 2010, Osman and Osman, 2013). The humid climate and low soil disturbance in Natural Forests promote SOC storage, while the low occurrence of wildfires prevents carbon loss (Chen *et al*., 2018). However, deforestation poses a threat, highlighting the importance of conservation efforts in enhancing SOC sequestration (Odebiri *et al*., 2023a).

### 4.2 Performance of the SOC DNN model

This study found that the DNN model’s accuracy in predicting SOC stocks decreases with soil depth. Specifically, model accuracy dropped from an R^2^ of 0.68 in the top 30cm (A horizon) to 0.53 in the next 30cm (B horizon), indicating that environmental covariates’ relationship with SOC stocks diminishes deeper in the soil. This observation aligns with previous research (Nussbaum *et al*., 2014, Li *et al*., 2023) and is possibly attributed to varying soil properties like texture, structure, and moisture at different depths (Liang *et al*., 1996, Coblinski *et al*., 2020). These changes reduce the correlation between environmental factors and SOC stocks, as confirmed by the higher SOC stock variance observed at deeper depths (Table 2). Furthermore, increasing soil depth amplifies the complexity of soil properties due to formation processes and biological impacts, affecting SOC relationships (Van Breemen and Buurman, 2002, Cornelis and Delvaux, 2016). For example, an increase in bulk density with depth decreases the pore space for microbial activity, weakening correlations between soil properties and SOC. This complexity, coupled with greater soil heterogeneity, may hinder precise SOC predictions, leading to higher errors and reduced model accuracy, a finding supported by Taghizadeh-Mehrjardi *et al*. (2016).

This study shows that the DNN model, although trained on legacy data, still exhibited a relatively high accuracy in topsoil SOC prediction (R^2^ = 0.68), attributed to the model’s layered structure. This highlights the necessity of integrating climate, topography, and soil attributes to predict SOC across various depths (Taghizadeh-Mehrjardi *et al*., 2016, Tayebi *et al*., 2021). Utilizing the SHAP technique, the research identified that the key predictors of SOC vary by depth, with rainfall, clay content, temperature, elevation, and RVI being crucial, albeit in differing orders, across depths (Figure 4). This variation underscores the complex interplay of environmental factors affecting SOC at different soil layers, reinforcing the critical need for comprehensive modelling that accounts for such diversity (Jobbágy and Jackson, 2000, Albaladejo *et al*., 2013).

Rainfall was found to have a significant impact on SOC stocks in the 0-60 cm depth, influencing litter input and decomposition, which aligns with previous studies (Sheikh *et al*., 2009, Blanco-Canqui *et al*., 2011). This is due to rainfall’s influence on biomass productivity, soil moisture, hydrological processes, vegetation density, and decomposition (D’odorico et al., 2003), which is crucial for SOC storage at this depth. Rainfall is also recognized as an indicator of water availability and soil moisture in different biomes and affects litter input and decomposition. Meanwhile, temperature plays a key role in SOC dynamics at deeper depths (Gross and Harrison, 2019), with lower temperatures decreasing microbial activity and promoting SOC accumulation (Ge *et al*., 2022). However, temperature variations can affect soil moisture levels and microbial activity differently across depths, influencing SOC storage (Grosse *et al*., 2011). However, this effect is not uniform across all depths, as other factors such as soil texture, water availability, and microbial community composition can play a more substantial role in SOC dynamics (Hamarashid *et al*., 2010).

Apart from climate, soil texture, particularly clay content, was found to be pivotal in determining SOC at various depths (Mao *et al*., 2015). Soil texture is determined by the relative amounts of sand, silt, and clay in a soil, and impacts several key soil properties that affect SOC storage and dynamics (Wiesmeier *et al*., 2019). In KZN, Clay and Alluvial soils are notable for their high nutrient content, excellent water retention capacity, and for promoting vegetation growth and SOC accumulation (Mucina and Rutherford, 2006, Fey, 2010). Soil with higher clay contents enhances SOC storage by increasing the surface area for organic matter adsorption, especially crucial in lower soil depths where water infiltration can mobilize dissolved organic matter (Wiesmeier *et al*., 2015). Conversely, Coastal sandy and Rocky soils in KZN, with low nutrient retention and high erosion rates, typically have lower SOC content due to limited water-holding capacity and organic matter (Mucina and Rutherford, 2006, Fey, 2010). Histosols, prevalent in coastal regions and wetlands in the province, hold significant SOC due to high organic matter content, with Batjes (1996) estimating that they hold approximately 65% of their SOC up to a depth of 2m. Thus, understanding soil texture is essential for assessing SOC at different depths since it offers insight into the potential for SOC storage in the soil and the conditions that influence the decomposition and mineralization of the organic matter.

Topography significantly affects SOC stocks, with elevation and slope influencing soil moisture, temperature, and vegetation (Liu *et al*., 2011), thereby impacting SOC content (Chen *et al*., 2016). Additionally, remote sensing data like Sentinel-1 VH and VV, and the RVI, play a crucial role in SOC estimation across soil depths. VH and VV, known for their soil moisture estimation capabilities, and RVI, sensitive to vegetation properties, help in estimating SOC, especially in topsoil where most organic matter accumulation occurs (Srivastava *et al*., 2006, Kornelsen and Coulibaly, 2013). Overall, remote sensing variables offer a valuable tool for estimating SOC at different soil depths by indirectly estimating factors that influence SOC accumulation and decomposition, highlighting the need for further research into their interplay and overall impact on SOC prediction (Sharma *et al*., 2022).

However, SOC stock models exhibit some uncertainty due to below-optimal profile datasets and inherent uncertainties in the data sources used (Owusu *et al*., 2020). Although legacy soil data presents opportunities for digital soil mapping in data-scarce regions, it also poses challenges resulting from its uneven spread and age, which can lead to wide prediction intervals of estimated SOC stocks (Owusu *et al*., 2020). Furthermore, the uneven distribution of data across land use types can impact the accuracy of predicted variables. To address these issues, future research could improve sampling schemes by targeting areas with wide prediction uncertainty and employ more advanced deep learning and remote sensing techniques to estimate SOC at different soil depths. Incorporating advances in remote sensing technology, such as combining Optical and Radar data with high spatial and spectral resolutions, as well as ancillary datasets, can provide detailed information necessary for predicting SOC content at deeper soil depths. Overall, integrating remote sensing and deep learning techniques holds great promise for advancing our understanding of SOC dynamics and improving the accuracy of SOC stock and flux estimation.

## 5 Conclusion

In conclusion, this study highlights the importance of reliably assessing SOC stocks at multiple depths across different vegetated landscape categories for effective climate change mitigation and sustainable soil management. The study implemented an advanced Deep Neural Network (DNN) model incorporating remote sensing data and soil physical properties to predict SOC stocks at various depths in the province of KwaZulu-Natal, South Africa. The results indicate a general decline in the predictive accuracy of the model with an increase in soil depth, underscoring the significance of integrating depth variations in soil properties when developing SOC models. Additionally, discernible factors were found to drive SOC stocks across distinct layers of topsoil, subsoil, and substratum, emphasizing the need for a comprehensive assessment of SOC stocks at various depths. Notably, Grasslands had the largest SOC stocks, while Commercial Forests demonstrated the greatest SOC sequestration capacity per unit area in KZN. These outcomes can facilitate the development of regional land management strategies that effectively tackle the ramifications of localized climate change in KZN. Future studies can build on the findings of this study by exploring the applicability of the developed DNN model in other regions with different soil properties, land-use categories, and climatic conditions. Moreover, to obtain a more comprehensive understanding of SOC stocks and its potential for sequestration, future research should investigate the influence of other environmental factors such as vegetation cover, soil moisture, and future land-use changes on SOC stocks at varying soil depths. Furthermore, exploring the impact of management practices such as tillage, cover cropping, and fertilization on SOC stocks can provide insights into effective soil management practices that promote SOC sequestration. Lastly, incorporating socioeconomic factors such as population growth and land tenure can help design management strategies that are socially, environmentally, and economically sustainable.

## Figures and Tables

**Table 1:** Predictors covariates and their description/formula.

Covariates	Description/formula	Reference	Temporal Period
			
**Sentinel 1 SAR data**			
VV Polarization	Ascending IW swath mode, 20m resolution	ESA	2014 – 2022 (medium image used)
VH Polarization	Ascending IW swath mode, 20m resolution	ESA	2014 – 2022 (medium image used)
Radar Vegetation Index (RVI)	4*VH/(VV+VH)	Kumar *et al.,* (2013)	2014 – 2022 (medium image used)
			
**Topo-climate**			
Elevation (DEM)	Ground height	Davy and Koen (2014)	2020
Slope	The steepness of the ground	Li *et al.,* (2014)	2020
Aspect	Slope direction	Rezaei and Gilkes, (2005)	2020
Topographic wetness index (TWI)	Steady state wetness index	Lang *et al.,* (2013)	2020
General curvature (Gen Curv)	Curvature both horizontally and vertically	Li *et al.,* (2014)	2020
Direct Insolation (Dir Ins)	Potential Incoming insolation	Rodriguez *et al.,* (2002)	2020
Profile curvature (Pro Curv)	Vertical rate of change of slope	Ritchie *et al.,* (2007)	2020
Catchment Area	Runoff velocity and volume	Kasai *et al.,* (2001)	2020
Rainfall	Mean annual precipitation	Odebiri *et al., *(2020b)	1960-2018
Temperature	Mean annual temperature	Odebiri *et al*., (2020b)	1960-2018
			
**Soil physical properties**			
Coarse fragments	Primary soil particle larger than 2 mm in nominal diameter	Hengl et al., (2017)	1950– June 2022
Clay content	Mineral particles smaller than 2 microns	Hengl et al., (2017)	1950– June 2022
Sand content	Natural granular material made up of finely divided rock and mineral particles	Hengl et al., (2017)	1950– June 2022
Silt content	Very small particles left as water sediment	Hengl et al., (2017)	1950– June 2022
Soil type	Soil classification based on the percentage of sand, silt, and clay in its composition.	FAO soil data portal	1950– June 2022

**Table 2:** Summary statistics of SOC stocks data (t/ha); new skewness and kurtosis indicated in brackets.

SOC depths(cm)	Min	Max	Mean	Standard deviation	Coefficient of variation (%)	Kurtosis	Skewness
0-30	26	149	57.53	16.84	29	5.9 (0.42)	1.4(0.10)
30-60	12	147	29.45	12.31	42	34.7(1.30)	4.0(0.19)
60-100	8	128	23.55	11.10	47	34.1(1.70)	4.2(0.44)
100-200	20	290	44.80	27.50	61	29.57(1.20)	4.2(0.60)

**Table 3:** Summary of the SOC stocks DNN model results at four different soil depths (0-30cm, 30-60cm, 60-100cm, and 100-200cm), for both the train and test data

SOC depths(t/h)	Train data			Test data		
	RMSE	R^2^		RMSE	R^2^	
0-30cm	7.34	0.74		8.34	0.68	
30-60cm	8.27	0.69		9.85	0.64	
60-100cm	10.59	0.59		13.75	0.53	
100-200cm	10.05	0.61		11.97	0.58	

**Table 4:** Soil organic carbon (SOC) content and stocks by each vegetated landscape (Grassland, Cropland, Natural Forest, and Commercial Forest) and soil depth (0-30 cm, 30-60 cm, 60-100 cm, and 100-200 cm). The table shows the area covered by each landscape, the total SOC stocks expressed in percentage, the minimum, mean, and maximum SOC content for each depth range. The significance letters (^a, b, c^) reflect statistically significant differences among land use groups for mean SOC based on Kruskal-Wallis and post-hoc testing. The letter ‘^a^’ implies that there is no major difference between commercial and natural forests. The letter ‘^b^’ applied to cropland shows no substantial difference from natural forest, but considerable differences from grassland and commercial forest. The letter ‘^c^’, peculiar to grassland, denotes substantial distinctions from all other categories. There were no significant changes noted in the minimum and maximum SOC levels among land uses at each depth.

Vegetated landscapes	Total SOC Area (%)	Min SOC (t/h)	Mean SOC(t/h)	Mean SOC Concentration (dg/kg)	Max SOC (t/h)
0-30cm SOC					
Grassland	46.36	26.56	53.81^ c^	182.80	144.45
Cropland	21.50	26.01	51.70^ b^	195.87	142.32
Natural forest	21.33	27.67	57.27^ a^	220.05	149.68
Commercial forest	10.81	28.69^c^	62.44 ^a^	259.17	147.00
					
**30-60cm SOC**					
Grassland	46.12	12.24	26.29^ c^	89.12	141.00
Cropland	21.71	12.11	22.65^ b^	105.26	105.93
Natural forest	21.66	15.01	29.12^ a^	111.36	147.99
Commercial forest	10.51	13.04	32.09^ a^	99.07	127.94
					
**60-100cm SOC**					
Grassland	46.02	8.17	21.62^ c^	57.91	128.00
Cropland	21.89	7.44	23.19^ b^	57.89	91.47
Natural forest	22.03	9.65	24.78^ a^	67.11	127.99
Commercial forest	10.06	8.04	25.71^ a^	68.09	111.05
					
**100-200cm SOC**					
Grassland	46.92	20.53	47.66^ c^	46.60	290.20
Cropland	21.65	20.50	47.32^ b^	42.10	206.55
Natural forest	21.17	22.04	47.12^ a^	50.13	289.98
Commercial forest	10.27	22.56	51.95^ a^	55.12	251.33

## Data Availability

Data are available from the authors upon request.

## References

[R1] Abera TA, Vuorinne I, Munyao M, Pellikka PK, Heiskanen J (2022). Land cover map for multifunctional landscapes of taita taveta county, kenya, based on sentinel-1 radar, sentinel-2 optical, and topoclimatic data. Data.

[R2] Albaladejo J, Ortiz R, Garcia-Franco N, Navarro AR, Almagro M, Pintado JG, Martínez-mena M (2013). Land use and climate change impacts on soil organic carbon stocks in semi-arid Spain. Journal of Soils and Sediments.

[R3] Alemu B (2014). The role of forest and soil carbon sequestrations on climate change mitigation. Res J Agr Environ Manage.

[R4] Allen M, Antwi-Agyei P, Aragon-Durand F, Babiker M, Bertoldi P, Bind M, Brown S, Buckeridge M, Camilloni I, Cartwright A (2019). Technical Summary: Global warming of 1.5 C An IPCC Special Report on the impacts of global warming of 1.5 C above pre-industrial levels and related global greenhouse gas emission pathways, in the context of strengthening the global response to the threat of climate change, sustainable development, and efforts to eradicate poverty.

[R5] Ampleman MD, Crawford KM, Fike DA (2014). Differential soil organic carbon storage at forb- and grass-dominated plant communities, 33 years after tallgrass prairie restoration. Plant and soil.

[R6] Angelopoulou T, Chabrillat S, Pignatti S, Milewski R, Karyotis K, Brell M, Ruhtz T, Bochtis D, Zalidis G (2023). Evaluation of airborne hyspex and spaceborne PRISMA hyperspectral remote sensing data for soil organic matter and carbonates estimation. Remote Sensing.

[R7] Assefa D, Rewald B, SandÉn H, Rosinger C, Abiyu A, Yitaferu B, Godbold DL (2017). Deforestation and land use strongly effect soil organic carbon and nitrogen stock in Northwest Ethiopia. Catena.

[R8] Avery B (1973). Soil classification in the Soil Survey of England and Wales. Journal of Soil Science.

[R9] Ayala Izurieta JE, Jara Santillán CA, Márquez CO, García VJ, Rivera-Caicedo JP, Van Wittenberghe S, Delegido J, Verrelst J (2022). Improving the remote estimation of soil organic carbon in complex ecosystems with Sentinel-2 and GIS using Gaussian processes regression. Plant and Soil.

[R10] Babaeian E, Sadeghi M, Jones SB, Montzka C, Vereecken H, Tuller M (2019). Ground, proximal, and satellite remote sensing of soil moisture. Reviews of Geophysics.

[R11] Bárcena T, Kiær L, Vesterdal L, Stefánsdóttir H, Gundersen P, Sigurdsson B (2014). Soil carbon stock change following afforestation in Northern Europe: a meta-analysis. Global Change Biology.

[R12] Batjes NH (1996). Total carbon and nitrogen in the soils of the world. European journal of soil science.

[R13] Batjes NH (2008). Mapping soil carbon stocks of Central Africa using SOTER. Geoderma.

[R14] Batjes NH, Ribeiro E, Van Oostrum A (2020). Standardised soil profile data to support global mapping and modelling (WoSIS snapshot 2019). Earth System Science Data.

[R15] Bishop T, Horta A, Karunaratne S (2015). Validation of digital soil maps at different spatial supports. Geoderma.

[R16] Blair J, Nippert J, Briggs J (2014). Grassland ecology 14. Ecology and the Environment.

[R17] Blanco-Canqui H, Schlegel A, Heer W (2011). Soil-profile distribution of carbon and associated properties in no-till along a precipitation gradient in the central Great Plains. Agriculture, ecosystems & environment.

[R18] Bot A, Benites J (2005). The importance of soil organic matter: Key to drought-resistant soil and sustained food production.

[R19] Briassoulis H (2020). Analysis of land use change: theoretical and modeling approaches.

[R20] Buchanan B, Fleming M, Schneider R, Richards B, Archibald J, Qiu Z, Walter M (2014). Evaluating topographic wetness indices across central New York agricultural landscapes. Hydrology and Earth System Sciences.

[R21] Carbutt C (2019). The Drakensberg Mountain Centre: A necessary revision of southern Africa’s high-elevation centre of plant endemism. South African Journal of Botany.

[R22] Cerri C, Easter M, Paustian K, Killian K, Coleman K, Bernoux M, Falloon P, Powlson D, Batjes N, Milne E (2007). Predicted soil organic carbon stocks and changes in the Brazilian Amazon between 2000 and 2030. Agriculture, ecosystems & environment.

[R23] Chai T, Draxler RR (2014). Root mean square error (RMSE) or mean absolute error (MAE). Geoscientific model development discussions.

[R24] Chaopricha NT, Marín-Spiotta E (2014). Soil burial contributes to deep soil organic carbon storage. Soil Biology and Biochemistry.

[R25] Chen S, Ai X, Dong T, Li B, Luo R, Ai Y, Chen Z, Li C (2016). The physico-chemical properties and structural characteristics of artificial soil for cut slope restoration in Southwestern China. Scientific Reports.

[R26] Chen S, Arrouays D, Angers DA, Martin MP, Walter C (2019). Soil carbon stocks under different land uses and the applicability of the soil carbon saturation concept. Soil and Tillage Research.

[R27] Chen S, Wang W, Xu W, Wang Y, Wan H, Chen D, Tang Z, Tang X, Zhou G, Xie Z (2018). Plant diversity enhances productivity and soil carbon storage. Proceedings of the National Academy of Sciences.

[R28] Chen X, Zhang D, Liang G, Qiu Q, Liu J, Zhou G, Liu S, Chu G, Yan J (2015). Effects of precipitation on soil organic carbon fractions in three subtropical forests in southern China. J plant ecol.

[R29] Coblinski JA, Giasson É, Demattê JA, Dotto AC, Costa JJF, Vašát R (2020). Prediction of soil texture classes through different wavelength regions of reflectance spectroscopy at various soil depths. Catena.

[R30] Cockburn J, Rouget M, Slotow R, Roberts D, Boon R, Douwes E, O’Donoghue S, Downs CT, Mukherjee S, Musakwa W (2016). How to build science-action partnerships for local land-use planning and management: lessons from Durban, South Africa. Ecology and Society.

[R31] Conant RT, Paustian K, Elliott ET (2001). Grassland management and conversion into grassland: effects on soil carbon. Ecological applications.

[R32] Cornelis JT, Delvaux B (2016). Soil processes drive the biological silicon feedback loop. Functional Ecology.

[R33] D’odorico P, Laio F, Porporato A, Rodriguez-Iturbe I (2003). Hydrologic controls on soil carbon and nitrogen cycles. II. A case study. Advances in Water Resources.

[R34] Dell B, Bartle J, Tacey W (1983). Root occupation and root channels of jarrah forest subsoils. Australian Journal of Botany.

[R35] Derenne S, Largeau C (2001). A review of some important families of refractory macromolecules: composition, origin, and fate in soils and sediments. Soil Science.

[R36] Dlamini P, Orchard C, Jewitt G, Lorentz S, Titshall L, Chaplot V (2011). Controlling factors of sheet erosion under degraded grasslands in the sloping lands of KwaZulu-Natal, South Africa. Agricultural Water Management.

[R37] Don A, Schumacher J, Freibauer A (2011). Impact of tropical land-use change on soil organic carbon stocks–a meta-analysis. Global Change Biology.

[R38] Dotto AC, Dalmolin RSD, Ten Caten A, Grunwald S (2018). A systematic study on the application of scatter-corrective and spectral-derivative preprocessing for multivariate prediction of soil organic carbon by Vis-NIR spectra. Geoderma.

[R39] Dovey SB (2014). Current carbon stock estimation capability for South African commercial forest plantations.

[R40] Edwards CA, Arancon NQ (2022). Biology and Ecology of Earthworms.

[R41] Eilers KG, Debenport S, Anderson S, Fierer N (2012). Digging deeper to find unique microbial communities: the strong effect of depth on the structure of bacterial and archaeal communities in soil. Soil Biology and Biochemistry.

[R42] Emadi M, Taghizadeh-Mehrjardi R, Cherati A, Danesh M, Mosavi A, Scholten T (2020). Predicting and mapping of soil organic carbon using machine learning algorithms in Northern Iran. Remote Sensing.

[R43] Esri AD (2011). Release 10 Documentation Manual.

[R44] Farrell HL, Barberán A, Danielson RE, Fehmi JS, Gornish ES (2020). Disturbance is more important than seeding or grazing in determining soil microbial communities in a semiarid grassland. Restoration Ecology.

[R45] Fey M (2010). Soils of South Africa.

[R46] Follett R (2001). Soil management concepts and carbon sequestration in cropland soils. Soil and Tillage Research.

[R47] Fornara D, Tilman D (2008). Plant functional composition influences rates of soil carbon and nitrogen accumulation. Journal of Ecology.

[R48] Fraser S (2012). South Africa: A Photographic Exploration of its People, Places & Wildlife.

[R49] Fuhrman J, Mcjeon H, Patel P, Doney SC, Shobe WM, Clarens AF (2020). Food–energy–water implications of negative emissions technologies in a+ 1.5 C future. Nature Climate Change.

[R50] Garnas J, Hurley B, Slippers B, Wingfield MJ, Roux J (2016). Insects and diseases of Mediterranean forests: A South African perspective. Insects and diseases of Mediterranean forest systems.

[R51] Ge J, Xu W, Xiong G, Zhao C, Li J, Liu Q, Tang Z, Xie Z (2022). Depth-dependent controls over soil organic carbon stock across Chinese shrublands. Ecosystems.

[R52] Germon A, Laclau J-P, Robin A, Jourdan C (2020). Tamm Review: Deep fine roots in forest ecosystems: Why dig deeper?. Forest Ecology and Management.

[R53] Ghosh P, Mahanta S (2014). Carbon sequestration in grassland systems. Range Management and Agroforestry.

[R54] Gomes AL, Revermann R, Gonçalves FM, Lages F, Aidar MP, Mostajo GAS, Finckh M (2021). Suffrutex grasslands in south-central Angola: belowground biomass, root structure, soil characteristics and vegetation dynamics of the ‘underground forests of Africa’. Journal of Tropical Ecology.

[R55] Gross CD, Harrison RB (2019). The case for digging deeper: soil organic carbon storage, dynamics, and controls in our changing world. Soil Systems.

[R56] Grosse G, Harden J, Turetsky M, Mcguire AD, Camill P, Tarnocai C, Frolking S, Schuur EA, Jorgenson T, Marchenko S (2011). Vulnerability of high-latitude soil organic carbon in North America to disturbance. Journal of Geophysical Research: Biogeosciences.

[R57] Grüneberg E, Ziche D, Wellbrock N (2014). Organic carbon stocks and sequestration rates of forest soils in G ermany. Global change biology.

[R58] Guo L, Fu P, Shi T, Chen Y, Zhang H, Meng R, Wang S (2020). Mapping field-scale soil organic carbon with unmanned aircraft system-acquired time series multispectral images. Soil and Tillage Research.

[R59] Gush MB, De Lange WJ, Dye PJ, Geldenhuys CJ (2015). Water use and socio-economic benefit of the biomass of indigenous trees (volume 1).

[R60] Hagle TM, Mitchell GE (1992). Goodness-of-fit measures for probit and logit. American Journal of Political Science.

[R61] Hamarashid NH, Othman MA, Hussain M-AH (2010). Effects of soil texture on chemical compositions, microbial populations and carbon mineralization in soil. Egypt J Exp Biol(Bot).

[R62] Harper AB, Powell T, Cox PM, House J, Huntingford C, Lenton TM, Sitch S, Burke E, Chadburn SE, Collins WJ (2018). Land-use emissions play a critical role in land-based mitigation for Paris climate targets. Nature communications.

[R63] He M, Tang L, Li C, Ren J, Zhang L, Li X (2022). Dynamics of soil organic carbon and nitrogen and their relations to hydrothermal variability in dryland. Journal of Environmental Management.

[R64] Heikkinen J, Keskinen R, Regina K, Honkanen H, Nuutinen V (2021). Estimation of carbon stocks in boreal cropland soils-methodological considerations. European Journal of Soil Science.

[R65] Hemati A, Nazari M, Asgari Lajayer B, Smith DL, Astatkie T (2022). Lignocellulosics in plant cell wall and their potential biological degradation. Folia Microbiologica.

[R66] Hengl T, Mendes De Jesus J, Heuvelink GB, Ruiperez Gonzalez M, Kilibarda M, Blagotić A, Shangguan W, Wright MN, Geng X, Bauer-Marschallinger B (2017). SoilGrids250m: Global gridded soil information based on machine learning. PLoS one.

[R67] Hitayezu P, Zegeye EW, Ortmann GF (2016). Farm-level crop diversification in the Midlands region of Kwazulu-Natal, South Africa: patterns, microeconomic drivers, and policy implications. Agroecology and Sustainable Food Systems.

[R68] Hobley EU, Wilson B (2016). The depth distribution of organic carbon in the soils of eastern Australia. Ecosphere.

[R69] Hou G, Delang CO, Lu X, Gao L (2009). Soil organic carbon storage varies with stand ages and soil depths following afforestation. Annals of Forest Research.

[R70] Hussain S, Hussain S, Guo R, Sarwar M, Ren X, Krstic D, Aslam Z, Zulifqar U, Rauf A, Hano C (2021). Carbon sequestration to avoid soil degradation: A review on the role of conservation tillage. Plants.

[R71] Jandl R, Lindner M, Vesterdal L, Bauwens B, Baritz R, Hagedorn F, Johnson DW, Minkkinen K, Byrne KA (2007). How strongly can forest management influence soil carbon sequestration?. Geoderma.

[R72] Jandl R, Rodeghiero M, Martinez C, Cotrufo MF, Bampa F, Van Wesemael B, Harrison RB, Guerrini IA, Richter DD, Rustad L (2014). Current status, uncertainty and future needs in soil organic carbon monitoring. Science of the total environment.

[R73] Jewitt D, Goodman PS, Erasmus BF, O’connor TG, Witkowski ET (2015). Systematic land-cover change in KwaZulu-Natal, South Africa: Implications for biodiversity. South African Journal of Science.

[R74] Jobbágy EG, Jackson RB (2000). The vertical distribution of soil organic carbon and its relation to climate and vegetation. Ecological applications.

[R75] John K, Abraham Isong I, Michael Kebonye N, Okon Ayito E, Chapman Agyeman P, Marcus Afu S (2020). Using machine learning algorithms to estimate soil organic carbon variability with environmental variables and soil nutrient indicators in an alluvial soil. Land.

[R76] Kalev SD, Toor GS (2018). Green Chemistry.

[R77] Kaye JP, Quemada M (2017). Using cover crops to mitigate and adapt to climate change. A review. Agronomy for sustainable development.

[R78] Kenye A, Sahoo UK, Singh SL, Gogoi A (2019). Soil organic carbon stock of different land uses of Mizoram, Northeast India. AIMS Geosciences.

[R79] Khumalo SA (2016). Environmental impact of household solid waste disposal practices on plant growth in rural areas of KwaZulu-Natal: A case study of UThukela District Municipality.

[R80] Kim T, Han H (2023). Coseismic displacement fields and the slip mechanism of the 2021 Mw 6.7 Hovsgol earthquake in Mongolia constrained by Sentinel-1 and ALOS-2 InSAR. GIScience & Remote Sensing.

[R81] Kokhanovsky A, Lamare M, Danne O, Brockmann C, Dumont M, Picard G, Arnaud L, Favier V, Jourdain B, Le Meur E (2019). Retrieval of snow properties from the Sentinel-3 Ocean and Land Colour Instrument. Remote Sensing.

[R82] Kornelsen KC, Coulibaly P (2013). Advances in soil moisture retrieval from synthetic aperture radar and hydrological applications. Journal of Hydrology.

[R83] Kukal S, Bawa S (2014). Soil organic carbon stock and fractions in relation to land use and soil depth in the degraded Shiwaliks hills of lower Himalayas. Land Degradation & Development.

[R84] Kumar D, Rao S, Sharma J (2013). Radar Vegetation Index as an alternative to NDVI for monitoring of soyabean and cotton.

[R85] Lai R (2004). Soil carbon sequestration in natural and managed tropical forest ecosystems. Journal of Sustainable Forestry.

[R86] Lal R (2004). Soil carbon sequestration in India. Climatic Change.

[R87] Lal R (2005). Forest soils and carbon sequestration. Forest ecology and management.

[R88] Lamichhane S, Adhikari K, Kumar L (2022). National soil organic carbon map of agricultural lands in Nepal. Geoderma Regional.

[R89] Lang M, Mccarty G, Oesterling R, Yeo I-Y (2013). Topographic metrics for improved mapping of forested wetlands. Wetlands.

[R90] Leblois A, Damette O, Wolfersberger J (2017). What has driven deforestation in developing countries since the 2000s? Evidence from new remote-sensing data. World Development.

[R91] Lee H, Lautenbach S, Nieto APG, Bondeau A, Cramer W, Geijzendorffer IR (2019). The impact of conservation farming practices on Mediterranean agro-ecosystem services provisioning—a meta-analysis. Regional Environmental Change.

[R92] Li H, Wu Y, Chen J, Zhao F, Wang F, Sun Y, Zhang G, Qiu L (2021a). Responses of soil organic carbon to climate change in the Qilian Mountains and its future projection. Journal of Hydrology.

[R93] Li J, Roy DP (2017). A global analysis of Sentinel-2A, Sentinel-2B and Landsat-8 data revisit intervals and implications for terrestrial monitoring. Remote Sensing.

[R94] Li X, Ding J, Liu J, Ge X, Zhang J (2021b). Digital Mapping of Soil Organic Carbon Using Sentinel Series Data: A Case Study of the Ebinur Lake Watershed in Xinjiang. Remote Sensing.

[R95] Li Y, Li Z, Cui S, Liang G, Zhang Q (2021c). Microbial-derived carbon components are critical for enhancing soil organic carbon in no-tillage croplands: A global perspective. Soil and Tillage Research.

[R96] Li Y, Wang X, Chen Y, Gong X, Yao C, Cao W, Lian J (2023). Application of predictor variables to support regression kriging for the spatial distribution of soil organic carbon stocks in native temperate grasslands. Journal of Soils and Sediments.

[R97] Liang X, Wood EF, Lettenmaier DP (1996). Surface soil moisture parameterization of the VIC-2L model: Evaluation and modification. Global and Planetary Change.

[R98] Libohova Z, Seybold C, Wysocki D, Wills S, Schoeneberger P, Williams C, Lindbo D, Stott D, Owens PR (2018). Reevaluating the effects of soil organic matter and other properties on available water-holding capacity using the National Cooperative Soil Survey Characterization Database. Journal of Soil and Water Conservation.

[R99] Lin B-J, Li R-C, Yang M-Y, Kan Z-R, Virk AL, Yves N, Bohoussou D, Zhao X, Dang YP, Zhang H-L (2023). Changes in cropland soil carbon through improved management practices in China: A meta-analysis. Journal of Environmental Management.

[R100] Liu X, Herbert S, Hashemi A, Zhang XF, Ding G (2006). Effects of agricultural management on soil organic matter and carbon transformation-a review. Plant Soil and Environment.

[R101] Liu Z, Shao MA, Wang Y (2011). Effect of environmental factors on regional soil organic carbon stocks across the Loess Plateau region, China. Agriculture, Ecosystems & Environment.

[R102] López-Mondéjar R, Zühlke D, Becher D, Riedel K, Baldrian P (2016). Cellulose and hemicellulose decomposition by forest soil bacteria proceeds by the action of structurally variable enzymatic systems. Scientific reports.

[R103] Lorenz K, Lal R (2005). The depth distribution of soil organic carbon in relation to land use and management and the potential of carbon sequestration in subsoil horizons. Advances in agronomy.

[R104] Lorenz K, Lal R (2014). Soil organic carbon sequestration in agroforestry systems. A review. Agronomy for Sustainable Development.

[R105] Lorenz K, Lal R (2015). Managing soil carbon stocks to enhance the resilience of urban ecosystems. Carbon Management.

[R106] Lorenz K, Lal R, Preston CM, Nierop KG (2007). Strengthening the soil organic carbon pool by increasing contributions from recalcitrant aliphatic bio (macro) molecules. Geoderma.

[R107] Louw JH (2016). The value of six key soil variables for incorporation into a South African forest site classification system. Southern Forests: a Journal of Forest Science.

[R108] Lundberg SM, Lee S-I (2017). A unified approach to interpreting model predictions. Advances in neural information processing systems.

[R109] Luo Z, Viscarra-Rossel RA, Qian T (2021). Similar importance of edaphic and climatic factors for controlling soil organic carbon stocks of the world. Biogeosciences.

[R110] Mandal D, Kumar V, Ratha D, Dey S, Bhattacharya A, Lopez-Sanchez JM, Mcnairn H, Rao YS (2020). Dual polarimetric radar vegetation index for crop growth monitoring using sentinel-1 SAR data. Remote Sensing of Environment.

[R111] Mao D, Wang Z, Li L, Miao Z, Ma W, Song C, Ren C, Jia M (2015). Soil organic carbon in the Sanjiang Plain of China: storage, distribution and controlling factors. Biogeosciences.

[R112] Mariappan S, David Raj A, Kumar S, Chatterjee U (2023). Ecological Footprints of Climate Change: Adaptive Approaches and Sustainability.

[R113] Marín-Spiotta E, Hobley EU (2022). DEEP SOIL CARBON. Multi-Scale Biogeochemical Processes in Soil Ecosystems: Critical Reactions and Resilience to Climate Changes.

[R114] Marín-Spiotta E, Sharma S (2013). Carbon storage in successional and plantation forest soils: a tropical analysis. Global Ecology and Biogeography.

[R115] Mashao FM, Mothapo MC, Munyai RB, Letsoalo JM, Mbokodo IL, Muofhe TP, Matsane W, Chikoore H (2023). Extreme Rainfall and Flood Risk Prediction over the East Coast of South Africa. Water.

[R116] Matinfar HR, Maghsodi Z, Mousavi SR, Rahmani A (2021). Evaluation and Prediction of Topsoil organic carbon using Machine learning and hybrid models at a Field-scale. Catena.

[R117] Matthews W, Van Wyk A, Van Rooyen N, Botha G (2001). Vegetation of the Tembe Elephant Park, Maputaland, South Africa. South African Journal of Botany.

[R118] Mbaabu PR, Olago D, Gichaba M, Eckert S, Eschen R, Oriaso S, Choge SK, Linders TEW, Schaffner U (2020). Restoration of degraded grasslands, but not invasion by Prosopis juliflora, avoids trade-offs between climate change mitigation and other ecosystem services. Scientific reports.

[R119] Mensah AK (2015). Role of revegetation in restoring fertility of degraded mined soils in Ghana: A review. International journal of biodiversity and conservation.

[R120] Merino AN, Fernández-López A, Solla-Gullón F, Edeso JM (2004). Soil changes and tree growth in intensively managed Pinus radiata in northern Spain. Forest ecology and management.

[R121] Moyo H, Slotow R, Rouget M, Mugwedi L, Douwes E, Tsvuura Z, Tshabalala T (2021). Adaptive management in restoration initiatives: Lessons learned from some of South Africa’s projects. South African Journal of Botany.

[R122] Mucina L, Rutherford MC (2006). The vegetation of South Africa, Lesotho and Swaziland.

[R123] Nagler T, Rott H, Hetzenecker M, Wuite J, Potin P (2015). The Sentinel-1 mission: New opportunities for ice sheet observations. Remote Sensing.

[R124] Nair PR, Nair VD, Kumar BM, Showalter JM (2010). Carbon sequestration in agroforestry systems. Advances in agronomy.

[R125] Nayak AK, Rahman MM, Naidu R, Dhal B, Swain CK, Nayak AD, Tripathi R, Shahid M, Islam MR, Pathak H (2019). Current and emerging methodologies for estimating carbon sequestration in agricultural soils: A review. Science of the total environment.

[R126] Ndlovu M, Clulow AD, Savage MJ, Nhamo L, Magidi J, Mabhaudhi T (2021). An assessment of the impacts of climate variability and change in KwaZulu-Natal Province, South Africa. Atmosphere.

[R127] Nell JP, Van Huyssteen CW (2014). Geology and groundwater regions to quantify primary salinity, sodicity and alkalinity in South African soils. South African Journal of Plant and Soil.

[R128] Ngo KM, Turner BL, Muller-Landau HC, Davies SJ, Larjavaara M, Bin Nik Hassan NF, Lum S (2013). Carbon stocks in primary and secondary tropical forests in Singapore. Forest Ecology and Management.

[R129] Norman N (2013). Geological journeys: A traveller’s guide to South Africa’s rocks and landforms.

[R130] Nussbaum M, Papritz A, Baltensweiler A, Walthert L (2014). Estimating soil organic carbon stocks of Swiss forest soils by robust external-drift kriging. Geoscientific Model Development.

[R131] O’Brien SL, Jastrow JD, Grimley DA, Gonzalez-Meler MA (2010). Moisture and vegetation controls on decadal-scale accrual of soil organic carbon and total nitrogen in restored grasslands. Global Change Biology.

[R132] Odebiri O, Mutanga O, Odindi J (2022a). Deep learning-based national scale soil organic carbon mapping with Sentinel-3 data. Geoderma.

[R133] Odebiri O, Mutanga O, Odindi J, Naicker R (2022b). Modelling soil organic carbon stock distribution across different land-uses in South Africa: A remote sensing and deep learning approach. ISPRS Journal of Photogrammetry and Remote Sensing.

[R134] Odebiri O, Mutanga O, Odindi J, Naicker R (2023a). Mapping soil organic carbon distribution across South Africa’s major biomes using remote sensing-topo-climatic covariates and Concrete Autoencoder-Deep neural networks. Science of The Total Environment.

[R135] Odebiri O, Mutanga O, Odindi J, Naicker R, Masemola C, Sibanda M (2021). Deep learning approaches in remote sensing of soil organic carbon: A review of utility, challenges, and prospects. Environmental monitoring and assessment.

[R136] Odebiri O, Mutanga O, Odindi J, Naicker R, Slotow R, Mngadi M (2023b). Evaluation of projected soil organic carbon stocks under future climate and land cover changes in South Africa using a deep learning approach. Journal of Environmental Management.

[R137] Odebiri O, Mutanga O, Odindi J, Peerbhay K, Dovey S, Ismail R (2020). Estimating soil organic carbon stocks under commercial forestry using topo-climate variables in KwaZulu-Natal, South Africa. South African Journal of Science.

[R138] Ontl T, Iversen C (2017). SPRUCE S1 bog areal coverage of hummock and hollow microtopography assessed along three transects in the S1 bog. ORNLTESSFA (Oak Ridge National Lab’s Terrestrial Ecosystem Science …).

[R139] Osman KT, Osman KT (2013). Forest soils.

[R140] Owusu S, Yigini Y, Olmedo GF, Omuto CT (2020). Spatial prediction of soil organic carbon stocks in Ghana using legacy data. Geoderma.

[R141] Padarian J, Mcbratney AB, Minasny B (2020). Game theory interpretation of digital soil mapping convolutional neural networks. Soil.

[R142] Palmer AR, Ainslie AM (2005). Grasslands of South Africa. Grasslands of the World.

[R143] Paul K, Polglase P, Nyakuengama J, Khanna P (2002). Change in soil carbon following afforestation. Forest ecology and management.

[R144] Pearson TR (2007). Measurement guidelines for the sequestration of forest carbon.

[R145] Peerbhay KY, Mutanga O, Ismail R (2013). Commercial tree species discrimination using airborne AISA Eagle hyperspectral imagery and partial least squares discriminant analysis (PLS-DA) in KwaZulu-Natal, South Africa. Isprs Journal of Photogrammetry and Remote Sensing.

[R146] Peter H, Jäggi A, Fernández J, Escobar D, Ayuga F, Arnold D, Wermuth M, Hackel S, Otten M, Simons W (2017). Sentinel-1A–First precise orbit determination results. Advances in space research.

[R147] Poeplau C, Don A, Vesterdal L, Leifeld J, Van Wesemael B, Schumacher J, Gensior A (2011). Temporal dynamics of soil organic carbon after land-use change in the temperate zone–carbon response functions as a model approach. Global change biology.

[R148] Pörtner H-O, Roberts DC, Adams H, Adler C, Aldunce P, Ali E, Begum RA, Betts R, Kerr RB, Biesbroek R (2022). Climate change 2022: Impacts, adaptation and vulnerability.

[R149] Price MH (2023). Mastering ArcGIS Pro.

[R150] Qiu F, Berglund J, Jensen JR, Thakkar P, Ren D (2004). Speckle noise reduction in SAR imagery using a local adaptive median filter. GIScience & Remote Sensing.

[R151] Rasmussen PE, Collins HP (1991). Long-term impacts of tillage, fertilizer, and crop residue on soil organic matter in temperate semiarid regions. Advances in agronomy.

[R152] Reynolds HL, Hungate BA, Chapin F, D’Antonio CM (1997). Soil heterogeneity and plant competition in anannual grassland. Ecology.

[R153] Rezaei SA, Gilkes RJ (2005). The effects of landscape attributes and plant community on soil chemical properties in rangelands. Geoderma.

[R154] Ritchie JC, Mccarty GW, Venteris ER, Kaspar T (2007). Soil and soil organic carbon redistribution on the landscape. Geomorphology.

[R155] Roberts D, Boon R, Diederichs N, Douwes E, Govender N, Mcinnes A, Mclean C, O’Donoghue S, Spires M (2012). Exploring ecosystem-based adaptation in Durban, South Africa:“learning-by-doing” at the local government coal face. Environment and Urbanization.

[R156] Rodríguez-Veiga P, Wheeler J, Louis V, Tansey K, Balzter H (2017). Quantifying forest biomass carbon stocks from space. Current Forestry Reports.

[R157] Rosinger C, Keiblinger K, Bieber M, Bernardini LG, Huber S, Mentler A, Sae-Tun O, Scharf B, Bodner G (2023). On-farm soil organic carbon sequestration potentials are dominated by site effects, not by management practices. Geoderma.

[R158] Roy KE (2016). Seeing the wood for the trees: an evaluation of the Buffelsdraai Landfill Community Reforestation Project.

[R159] Rumpel C, Chabbi A, Marschner B (2012). Carbon storage and sequestration in subsoil horizons: Knowledge, gaps and potentials. Recarbonization of the biosphere: ecosystems and the global carbon cycle.

[R160] Sahoo UK, Singh SL, Gogoi A, Kenye A, Sahoo SS (2019). Active and passive soil organic carbon pools as affected by different land use types in Mizoram, Northeast India. PloS one.

[R161] Sainepo BM, Gachene CK, Karuma A (2018). Assessment of soil organic carbon fractions and carbon management index under different land use types in Olesharo Catchment, Narok County, Kenya. Carbon balance and management.

[R162] Saiz G, Bird MI, Domingues T, Schrodt F, Schwarz M, Feldpausch TR, Veenendaal E, Djagbletey G, Hien F, Compaore H (2012). Variation in soil carbon stocks and their determinants across a precipitation gradient in W est A frica. Global change biology.

[R163] Scharlemann JP, Tanner EV, Hiederer R, Kapos V (2014). Global soil carbon: understanding and managing the largest terrestrial carbon pool. Carbon Management.

[R164] Shafizadeh-Moghadam H, Minaei F, Talebi-Khiyavi H, Xu T, Homaee M (2022). Synergetic use of multi-temporal Sentinel-1, Sentinel-2, NDVI, and topographic factors for estimating soil organic carbon. Catena.

[R165] Sharma R, Mishra DR, Levi MR, Sutter LA (2022). Remote Sensing of Surface and Subsurface Soil Organic Carbon in Tidal Wetlands: A Review and Ideas for Future Research. Remote Sensing.

[R166] Sheikh MA, Kumar M, Bussmann RW (2009). Altitudinal variation in soil organic carbon stock in coniferous subtropical and broadleaf temperate forests in Garhwal Himalaya. Carbon balance and management.

[R167] Singh P, Diwakar M, Shankar A, Shree R, Kumar M (2021). A Review on SAR Image and its Despeckling. Archives of Computational Methods in Engineering.

[R168] Smit C, Bredenkamp G, Van Rooyen N (1995). The grassland vegetation of the Low Drakensberg escarpment in the north-westernKwaZulu-Natal and north-eastern Orange Free State border area. South African Journal of Botany.

[R169] Song X-D, Wu H-Y, Ju B, Liu F, Yang F, Li D-C, Zhao Y-G, Yang J-L, Zhang G-L (2020). Pedoclimatic zone-based three-dimensional soil organic carbon mapping in China. Geoderma.

[R170] Sothe C, Gonsamo A, Arabian J, Kurz WA, Finkelstein SA, Snider J (2022). Large soil carbon storage in terrestrial ecosystems of Canada. Global Biogeochemical Cycles.

[R171] Souchère V, Cerdan O, Ludwig B, Le Bissonnais Y, Couturier A, Papy F (2003). Modelling ephemeral gully erosion in small cultivated catchments. Catena.

[R172] Srivastava HS, Patel P, Navalgund RR (2006). Microwave Remote Sensing of the Atmosphere and Environment V.

[R173] Taghizadeh-Mehrjardi R, Nabiollahi K, Kerry R (2016). Digital mapping of soil organic carbon at multiple depths using different data mining techniques in Baneh region, Iran. Geoderma.

[R174] Taghizadeh-Mehrjardi R, Schmidt K, Amirian-Chakan A, Rentschler T, Zeraatpisheh M, Sarmadian F, Valavi R, Davatgar N, Behrens T, Scholten T (2020). Improving the spatial prediction of soil organic carbon content in two contrasting climatic regions by stacking machine learning models and rescanning covariate space. Remote Sensing.

[R175] Tayebi M, Rosas FIM, Mendes WDS, Poppiel RR, Ostovari Y, Ruiz LFC, Santos DOS, Cerri CEP, Silva SHG, Curi N (2021). Drivers of organic carbon stocks in different LULC history and along soil depth for a 30 years image time series. Remote Sensing.

[R176] Tiefenbacher A, Sandén T, Haslmayr H-P, Miloczki J, Wenzel W, Spiegel H (2021). Optimizing carbon sequestration in croplands: a synthesis. Agronomy.

[R177] Tripathi A, Tiwari RK (2022). Utilisation of spaceborne C-band dual pol Sentinel-1 SAR data for simplified regression-based soil organic carbon estimation in Rupnagar, Punjab, India. Advances in Space Research.

[R178] Vågen TG, Lal R, Singh B (2005). Soil carbon sequestration in sub-Saharan Africa: a review. Land degradation & development.

[R179] Van Breemen N, Buurman P (2002). Soil formation.

[R180] Van Der Sande MT, Powers JS, Kuyper TW, Norden N, Salgado-Negret B, De Almeida SILVA, Bongers F, Delgado D, Dent DH, Derroire G (2023). Soil resistance and recovery during neotropical forest succession. Philosophical Transactions of the Royal Society B.

[R181] Vaudour E, Gomez C, Fouad Y, Lagacherie P (2019a). Sentinel-2 image capacities to predict common topsoil properties of temperate and Mediterranean agroecosystems. Remote Sensing of Environment.

[R182] Vaudour E, Gomez C, Loiseau T, Baghdadi N, Loubet B, Arrouays D, Ali L, Lagacherie P (2019b). The impact of acquisition date on the prediction performance of topsoil organic carbon from Sentinel-2 for croplands. Remote Sensing.

[R183] Venter ZS, Hawkins H-J, Cramer MD, Mills AJ (2021). Mapping soil organic carbon stocks and trends with satellite-driven high resolution maps over South Africa. Science of The Total Environment.

[R184] Wang B, Waters C, Orgill S, Gray J, Cowie A, Clark A, Li Liu D (2018). High resolution mapping of soil organic carbon stocks using remote sensing variables in the semi-arid rangelands of eastern Australia. Science of the Total Environment.

[R185] Wang D, Wu T, Zhao L, Mu C, Li R, Wei X, Hu G, Zou D, Zhu X, Chen J (2021a). A 1 km resolution soil organic carbon dataset for frozen ground in the Third Pole. Earth System Science Data.

[R186] Wang H, Zhang X, Wu W, Liu H (2021b). Prediction of Soil Organic Carbon under Different Land Use Types Using Sentinel-1/-2 Data in a Small Watershed. Remote Sensing.

[R187] Wang X, Dai W, Filley TR, Wang C, Bai E (2021c). Aboveground litter addition for five years changes the chemical composition of soil organic matter in a temperate deciduous forest. Soil Biology and Biochemistry.

[R188] Wang Y, Han X, Jin Z, Zhang C, Fang L (2016). Soil organic carbon stocks in deep soils at a watershed scale on the Chinese Loess Plateau. Soil Science Society of America Journal.

[R189] Wang Y, Zhang Z, Feng L, Du Q, Runge T (2020). Combining Multi-Source Data and Machine Learning Approaches to Predict Winter Wheat Yield in the Conterminous United States. Remote Sensing.

[R190] Ward SE, Smart SM, Quirk H, Tallowin JR, Mortimer SR, Shiel RS, Wilby A, Bardgett RD (2016). Legacy effects of grassland management on soil carbon to depth. Global change biology.

[R191] Wiesmeier M, Hübner R, Spörlein P, Geuß U, Hangen E, Reischl A, Schilling B, Von Lützow M, Kögel-Knabner I (2014). Carbon sequestration potential of soils in southeast Germany derived from stable soil organic carbon saturation. Global change biology.

[R192] Wiesmeier M, Munro S, Barthold F, Steffens M, Schad P, KÖgel-Knabner I (2015). Carbon storage capacity of semi-arid grassland soils and sequestration potentials in northern China. Global Change Biology.

[R193] Wiesmeier M, Urbanski L, Hobley E, Lang B, Von Lützow M, Marin-Spiotta E, Van Wesemael B, Rabot E, Ließ M, Garcia-Franco N (2019). Soil organic carbon storage as a key function of soils-A review of drivers and indicators at various scales. Geoderma.

[R194] Wu F, Li F, Zhao X, Bolan NS, Fu P, Lam SS, Mašek O, Ong HC, Pan B, Qiu X (2022). Meet the challenges in the “Carbon Age”. Carbon Research.

[R195] Xu S, Liu L, Sayer EJ (2013). Variability of above-ground litter inputs alters soil physicochemical and biological processes: a meta-analysis of litterfall-manipulation experiments. Biogeosciences.

[R196] Yang L, Cai Y, Zhang L, Guo M, Li A, Zhou C (2021). A deep learning method to predict soil organic carbon content at a regional scale using satellite-based phenology variables. International Journal of Applied Earth Observation and Geoinformation.

[R197] Yang M, Liu B, Zou J, Meng F, Wang F (2019). Advances in soil organic carbon mapping with hyperspectral remote sensing: A review of challenges and opportunities. Journal of Cleaner Production.

[R198] Zaloumis NP, Bond WJ (2016). Reforestation or conservation? The attributes of old growth grasslands in South Africa. Philosophical Transactions of the Royal Society B: Biological Sciences.

[R199] Zanella A, Bolzonella C, Lowenfels J, Ponge J-F, BouchÉ M, Saha D, Kukal SS, Fritz I, Savory A, Blouin M (2018). Humusica 2, article 19: Techno humus systems and global change–conservation agriculture and 4/1000 proposal. Applied Soil Ecology.

[R200] Zhang Y, Li Z, Li Y, Wu Y, Liu J (2020). Estimating soil organic carbon content in cropland using Sentinel-2 imagery and random forest algorithm. Journal of Applied Remote Sensing.

[R201] Zhou G, Guan L, Wei X, Tang X, Liu S, Liu J, Zhang D, Yan J (2008). Factors influencing leaf litter decomposition: an intersite decomposition experiment across China. Plant soil.

[R202] Zhou T, Geng Y, Lv W, Xiao S, Zhang P, Xu X, Chen J, Wu Z, Pan J, Si B (2023). Effects of optical and radar satellite observations within Google Earth Engine on soil organic carbon prediction models in Spain. Journal of Environmental Management.

[R203] Zhou X, Wen Y, Goodale UM, Zuo H, Zhu H, Li X, You Y, Yan L, Su Y, Huang X (2017). Optimal rotation length for carbon sequestration in Eucalyptus plantations in subtropical China. New Forests.

[R204] Zhuo Z, Chen Q, Zhang X, Chen S, Gou Y, Sun Z, Huang Y, Shi Z (2022). Soil organic carbon storage, distribution, and influencing factors at different depths in the dryland farming regions of Northeast and North China. Catena.

